# Unraveling Epigenetic Interplay between Inflammation, Thrombosis, and Immune-Related Disorders through a Network Meta-analysis

**DOI:** 10.1055/a-2222-9126

**Published:** 2024-02-02

**Authors:** Shankar Chanchal, Swati Sharma, Syed Mohd, Armiya Sultan, Aastha Mishra, Mohammad Zahid Ashraf

**Affiliations:** 1 Department of Biotechnology, Faculty of Natural Sciences, Jamia Millia Islamia, Delhi, India; 2 Cardio Respiratory Disease unit, CSIR- Institute of Genomics and Integrative Biology, Delhi, India

**Keywords:** thrombosis, inflammation, hypoxia, epigenetic modulators, miRNA, meta-analysis

## Abstract

Inflammation and thrombosis are two distinct yet interdependent physiological processes. The inflammation results in the activation of the coagulation system that directs the immune system and its activation, resulting in the initiation of the pathophysiology of thrombosis, a process termed immune-thrombosis. Still, the shared underlying molecular mechanism related to the immune system and coagulation has not yet been explored extensively. Inspired to answer this, we carried out a comprehensive gene expression meta-analysis using publicly available datasets of four diseases, including venous thrombosis, systemic lupus erythematosus, rheumatoid arthritis, and inflammatory bowel disease. A total of 609 differentially expressed genes (DEGs) shared by all four datasets were identified based on the combined effect size approach. The pathway enrichment analysis of the DEGs showed enrichment of various epigenetic pathways such as histone-modifying enzymes, posttranslational protein modification, chromatin organization, chromatin-modifying enzymes, HATs acetylate proteins. Network-based protein–protein interaction analysis showed epigenetic enzyme coding genes dominating among the top hub genes. The miRNA-interacting partner of the top 10 hub genes was determined. The predomination of epitranscriptomics regulation opens a layout for the meta-analysis of miRNA datasets of the same four diseases. We identified 30 DEmiRs shared by these diseases. There were 9 common DEmiRs selected from the list of miRNA-interacting partners of top 10 hub genes and shared significant DEmiRs from microRNAs dataset acquisition. These common DEmiRs were found to regulate genes involved in epigenetic modulation and indicate a promising epigenetic aspect that needs to be explored for future molecular studies in the context of immunothrombosis and inflammatory disease.

## Introduction

The human body invokes inflammatory responses to various exogenous and endogenous stimuli. Exogenous stimuli involve invasion by pathogens and environmental conditions such as temperature, hypoxia, and high ultraviolet radiation along with others. 1 2 3 Endogenous stimuli range from damaged cells released from diseased conditions to circulatory signaling molecules elicited by epigenetic changes. 4 Often the complex inflammatory process involving the interplay of immune cells, blood vessels, and various molecular mediators against these stimuli overlaps. Inflammation is interlinked with hemostasis and altered hemostasis is associated with several diseased conditions. 5 Any disturbance or loss in the control of hemostasis results in the amplification of inflammation contributing to the onset and manifestation of pathologies such as thrombosis. Inflammation is a key player in a thrombus or blood clot formation within the blood vessel via activation of the coagulation system. 5 Inflammation induces coagulation while coagulation amplifies inflammation. 6 However, the underlying mechanism through which inflammation invokes thrombosis remains under-explained and complicated. There are complex interactions between inflammation and coagulation pathways, involving pro-inflammatory cytokines, chemokines, tissue factor expression, adhesion molecules, endothelial cells, immune cells, and leukocyte activation along with platelet activation and aggregation. Such interactions further contribute to endothelial injury and dysfunction. 7 Previous work from our lab demonstrated the association between nucleotide-binding domain, leucine-rich containing family, pyrin domain containing 3 (NLRP3) inflammasome, and hypoxia-inducible factor 1-alpha (HIF-1α) in potentiating thrombosis under hypoxic conditions. 3 The study revealed that inflammation precedes coagulation in thrombosis under a hypoxic environment with a concomitant increase in the relative expression of NLRP3, caspase-1, interleukin-1β (IL-1β), and IL-18 transcripts in high altitude (HA) thrombotic patients. The involvement of inflammasome in the pathophysiology of several inflammatory disorders such as systemic lupus erythematosus (SLE), rheumatoid arthritis (RA), and inflammatory bowel disease (IBD) with prominent pro-thrombotic phenotypic features further indicates a strong link of interaction between inflammation and coagulation pathways. 7 These inflammatory disorders with explicit pro-thrombotic phenotypic features give an opportunity to understand the pathogenic influence and the active pathways that connect inflammation and coagulation pathways. This has encouraged us to look for the shared genetic cues in inflammatory diseases such as SLE, RA, and IBD along with venous thrombosis (VT).

SLE is an acquired, multi-organ and autoimmune disorder with diverse clinical manifestations including thrombosis prevalent in more than 10 percent of cases. 8 The incidences of thrombosis may even exceed up to 37% in high-risk SLE patients. 8 The exact etiology of thrombotic events in SLE remains obscure but various environmental, genetic, and hormonal factors contribute to its pathogenesis. The antiphospholipid syndrome in approximately a third of SLE patients favors thrombosis due to the endothelial injury and increased expression of intercellular adhesion molecule/vascular cell adhesion molecule, vascular endothelial growth factor, von Willebrand factor, and platelet activation. 9 Similarly, RA is a systemic inflammatory autoimmune disease characterized by persistent inflammation of multiple synovial joints. The incidence of thromboembolic events in RA patients can range from 30 to 50% compared to the general population because of predisposing conditions like endothelial dysfunction and hypercoagulability. RA patients also have the presence of pro-thrombotic milieu with increased fibrinogen level, protein C, protein S, and inflammatory markers and decreased antithrombin III. In addition, endothelial cells may be injured because of high levels of plasma homocysteine. 10 Sharing the same pathology of inflammation, IBD is a systemic disorder predominantly affecting the gastrointestinal tract along with several extra-intestinal manifestations including thrombosis. Bargen and Barker were the first to report the possible association between IBD and VT at the Mayo Clinic showing 18 patients with VT from among more than 1,000 patients treated for IBD. 11 Various other reports showed that patients with IBD have an increased risk of VT. 12 13

Although the thrombotic risk is well ascertained in all the above inflammatory diseases, but the evidence related to the hierarchical relationship between the biological process and shared pathways still is lacking. Therefore, in the present study, we carried out an integrated gene expression meta-analysis of four independent publicly available microarray datasets of the four selected diseases viz. VT, SLE, RA, and IBD, to identify shared gene expression signatures and overlapping biological pathways. We selected four eligible datasets to out the common transcriptional signatures based on the inclusion criteria from public repositories such as Gene Expression Omnibus (GEO) and ArrayExpress. Network-based hub gene analysis obtained an overrepresentation of chromatin modulators among the top enriched pathways and histone-modifying enzymes among the top identified hub genes. The dominance of epigenetic modulators in our transcriptomics meta-analysis made us examine quantitative and qualitative epitranscriptomics profiles targeting microRNAs (miRNAs) as well in the selected pathologies of the study.

Dysregulated expression of miRNAs augments the pathogenesis of several diseases. Therefore, by identifying the common miRNA signatures to summarize the complex multilevel regulation of miRNA and mRNA expression, in relation to disease, the body's physiological state and various external factors such as hypoxia become pertinent. As a result, shared genetics and epigenetics signatures and overlapping biological pathways are identified through meta-analysis of integrated transcriptomics and epitranscriptomics targeting miRNAs for four independent publicly available microarray datasets of VT, SLE, RA, and IBD.

## Materials and Methods

### Acquisition of Eligible Datasets for Meta-analysis

We systematically mined the public database National Centre for Biotechnology Information-GEO (NCBI-GEO) database ( http://www.ncbi.nlm.nih.gov/geo/ ) to select microarray-based studies. For search, following keywords and their combinations were used: “Thrombosis,” “venous thrombosis (VT),” “inflammatory disease,” “Systemic lupus erythematosus (SLE),” “Rheumatoid arthritis (RA),” “Inflammatory bowel disease (IBD),” “microarray,” “gene expression dataset.” Each identified study was used to extract the following information: GEO accession number, sample source, platform, number of controls, and patient along with references. For each dataset selection inclusion, criteria were laid and strictly followed. The criteria were human control/patient study, sample source, platform, entry type having datasets, and study type with expression profiling by array.

**Fig. 1:**
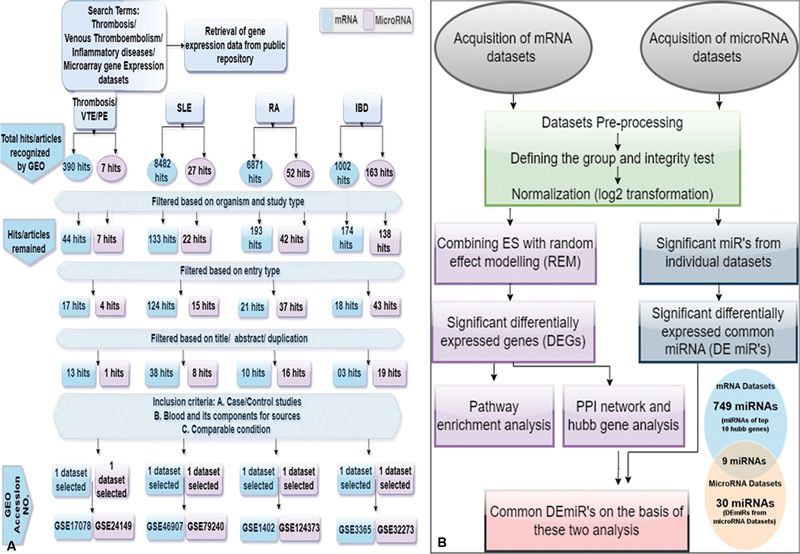
Workflow and processing of mRNA and microRNA microarray datasets. ( **A** ) Diagram depicting the workflow of the retrieval and selection of the microarray datasets along with inclusion and exclusion criteria of individual datasets included in the meta-analysis. ( **B** ) Workflow of the process of microarray and microRNA datasets through meta-analysis. Depiction of the flow chart of the process involved in integrated meta-analysis of selected microarray datasets of mRNA and miRNA expression. BOEC, blood outgrowth endothelial cells; GEO, gene expression omnibus; IBD, inflammatory bowel disease; PBMCs, peripheral blood mononuclear cells; PE, pulmonary embolism; RA, rheumatoid arthritis; SLE, systemic lupus erythematosus; VT, venous thrombosis.

We fetched the expression data from RNA extracted from the blood samples in all the conditions. However, when data were extracted for RA (GSE1402), we found that few samples had the gene expression data of synovial fluid mononuclear cell samples. So, we carefully excluded those samples in this dataset as they did not meet our inclusion criteria. The meta-analysis was conducted in accordance with the guidelines provided in the Preferred Reporting Items for Systematic Reviews and Meta-Analysis (PRISMA) guidelines published in 2009. The same exercise was conducted for microRNA datasets as well for the four studied pathologies. A flow diagram depicting the microarray meta-analysis as a selection process along with inclusion and exclusion criteria of eligible microarray datasets is represented in Fig. 1A .

### Batch Effect Correction and Preprocessing of Individual Datasets of Microarray

Integrative Meta-Analysis of Expression Data (INMEX), a web interface for integrative meta-analysis ( http://www.networkanalyst.ca/faces/home.xhtml ) tool was used for preprocessing and normalization of individual datasets using log2transformation with quantile autoscaling. 14 All gene and probe IDs were annotated by converting them to their corresponding Entrez IDs. The batch effect correction option was applied before performing the meta-analysis, to reduce the potential study-specific batch effect heterogeneity using the combat procedure of the INMEX tool. This step is mandatory for integrative analysis and reducing contradictory factors due to nonbiological variation. We performed principal component analysis (PCA) to visualize the sample clustering patterns before and after applying the ComBat procedure. It was done to ensure identical distribution among the samples. 15 Using empirical Bayes methods, the ComBat procedures stabilize the expression of genes with too-high or too-low ratios along with stabilizing the individual gene variances by shrinking variances across all the other genes.

### Microarray Meta-analysis: Discerning Shared Differentially Expressed Genes

INMEX was used to independently perform the differential expression analysis for each dataset using an adjusted *p* -value <0.05. The analysis was done based on the false discovery rate (FDR) using the Benjamini–Hochberg procedure and moderated *t* -test based on the Limma algorithm. 16 While conducting the meta-analysis, data integrity was checked to confirm the consistency and completeness of class labels across all the datasets. We conducted the differential expression meta-analysis across the diseased patients and healthy individuals (controls) by the random effects model (REM) based on combining the effect sizes (ESs). The analysis was done based on changes in gene expression from the different studies to obtain an overall mean with a significance level of *p* -value <0.05. 17 To avoid significant cross-study heterogeneities based on the Cochran's Q test, REM was chosen over the fixed effects model. 18

### Biological Process Analysis Using Kobas3.0

To explore the biological functions and reveal the significant and enriched pathway analysis of the shared differentially expressed genes (DEGs) of the above-mentioned diseases, an online user-friendly platform “Kobas3.0” ( http://kobas.cbi.pku.edu.cn/kobas3/?t=1 ), was used under the option of “Gene list Enrichment” including Gene Ontology (GO), KEGG, Reactome, PANTHER, and few other pathway analyses. 19 Kobas3.0 implements hypergeometric test/Fisher's exact test as a statistical test method with Benjamini–Hochberg as FDR correction method.

### Network-Based Hub Gene Analysis and Gene miRNA Interaction of the Shared DEGs

NetworkAnalyst/INMEX was used for network-based analysis for generating a protein–protein interaction (PPI) network depicting generic PPI and gene–miRNA interaction. A compiled list of DEGs was uploaded to the web-based server of NetworkAnalyst. To allow the proper visualization of the interaction network and avoid the “hairball effect,” the generic PPI network construction was restricted to contain only the original seed proteins and minimum associated protein by selecting the minimum network interactors and the obtained result was used for hub gene analysis by the Cytoscape tool, giving detailed information on nodes within the current network, including degree and betweenness centrality. 19 The gene–miRNA interaction network was also obtained similarly using INMEX by miRTarBase database and was restricted to a minimum network and fed into Cytoscape. The degree and betweenness centrality were explained as the number of connections to the other nodes and the number of shortest paths going through a node, respectively. 20 Fig. 1B depicts the complete workflow of the process followed throughout the analysis from acquiring the dataset to determining the miRNAs of the hub genes.

### Functional Enrichment Analysis of Common DEGs in between Inflammatory Diseases and Thrombosis

To understand the nexus of the shared DEGs on inflammatory diseases and thrombosis, a functional analysis was performed using ClueGO, a user-friendly Cytoscape plug-in. It was utilized to gain insights into a functionally grouped network of an enriched biological pathway on the shared DEGs. 21 The minimum interaction network with 760 nodes and 2,144 edges was downloaded from the INMEX and fed into the Cytoscape with their expression values and additionally, the names of the top 50 hub genes were provided to ClueGo for exploring the enriched and significant pathways and biological terms related to our networks of DEGs. The top 50 hub genes were specifically shortlisted based on the high degree of connectivity in the PPI network for functional analysis. ClueGO as a tool examines the interrelations of terms and functional groups in biological networks. It enables the user to make various flexible adjustments for a profound exploration of gene clusters in biological networks. The nonredundant biological terms for large clusters of genes/pathways obtained from functional enrichment analysis in a grouped network can be visualized by it. Enrichment (right-sided) hypergeometric distribution tests were used in the present study. The GO terms and pathways were ranked based on their significance with a cutoff *p* -value ≤0.05, followed by the Bonferroni adjustment for the terms. Using the Biological Networks Gene Ontology (BiNGO) tool, 22 an open-source Cytoscape plug-in to assess the overrepresentation of GO, we verified which GO biological process terms are significantly overrepresented in a set of DEGs by hyper-geometric test statistics, followed by Benjamini–Hochberg FDR correction. Heatmap visualization of the chromatin organization pathway of the DEGs from the meta-analysis was performed using the “Pattern extractor” tool from INMEX data for this heatmap normalized within each study before being pooled together.

### Processing and Screening of DEmiRs

After selecting desirable miRNA datasets, they were processed independently in the GEO2R ( http://www.ncbi.nlm.nih.gov/geo/geo2r/ ) tool, a web tool of the GEO repository, NCBI. GEO2R is an R-based platform used to perform comparisons between different groups of samples in each GEO dataset. DEmiRs were screened using a *p* -value of less than 0.05 as the threshold. After processing each miR dataset, they were compared with other datasets in a combinatorial method to find the common DEmiRs with the help of online Bioinformatics and Research computing website tools provided by Massachusetts Institute of Technology (MIT; http://barc.wi.mit.edu/tools/compare/index.php ). By comparing each miR dataset, the common DEmiRs in all the pathologies from the analysis are compared to miRNAs obtained computationally through mRNA expression via hub gene analysis. From the comparison, the obtained DEmiRs were subjected to Cytoscape to see the interaction of common DEmiRs with their target hub genes.

### Statistical Analyses

For conducting the meta-analysis, we adopted the ES combination using the REM by a web-based tool, INMEX. An adjusted *p* -value of <0.05, based on the FDR using the Benjamini–Hochberg procedure, was used to obtain and select shared DEGs. For the functional enrichment analysis, the hypergeometric test (right-sided) and Benjamini–Hochberg FDR correction with an adjusted *p* -value ≤0.05 were used. Important enriched pathways were identified using a hypergeometric test/Fisher's exact test as a statistical test method with Benjamini–Hochberg as the FDR correction method.

## Results

### Batch Effect Removal of Eligible Dataset Selection

A total of four microarray studies met the inclusion criteria and were selected further for the meta-analysis. The datasets for VT and datasets for inflammatory diseases, viz. SLE, RA, and IBD were included in the studies as mentioned in Fig. 1A . The publicly available microarray gene expression datasets related to VT, SLE, RA, and IBD were mined on NCBIGEO. On initial search, we found 390 hits for VT, 8,482 hits for SLE, 6,971 hits for RA, and 1,002 hits for IBD. After filtering each hit on the basis of organism and study type, we were left with 44 hits of VT, 133 hits for SLE, 193 hits for RA, and 174 hits for IBD. Then, the remaining hits were filtered based on the entry type. Overall, 17 hits fit for VT, 124 hits for SLE, 21 for RA, and 18 for IBD remained. Again, the remaining articles were filtered on the basis of title or abstract duplication, and we are left with 13 articles of VT, 38 articles of SLE, 10 articles of RA, and 3 articles of IBD. We finally filtered the remaining datasets on the basis of our inclusion criteria, i.e., healthy/patient studies having blood and its comparable as a source and a comparable condition in each condition. We finally observed one gene expression dataset fit for each disease for performing meta-analysis in accordance with all the above-mentioned criteria.

**Fig. 2:**
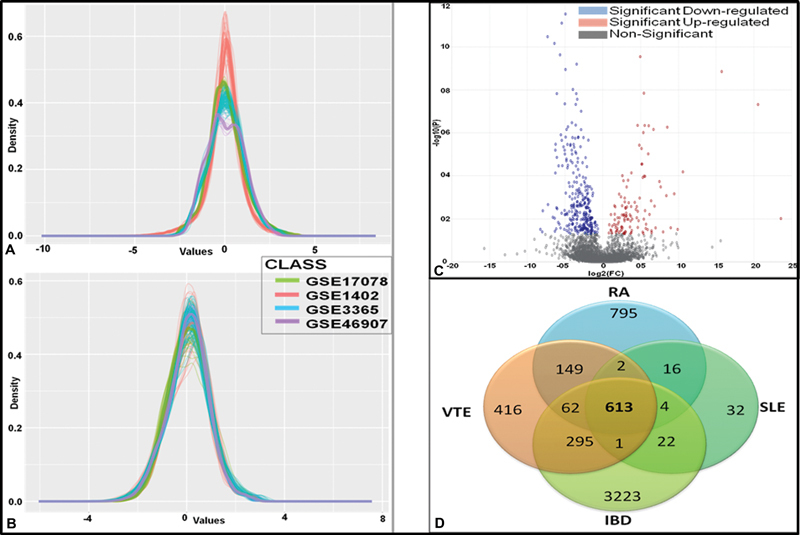
Analysis of datasets for the identification of gene and its share with other disease. ( **A** ) Density plot to compare clustering and distribution patterns before batch removal and ( **B** ) after applying batch removal using the Combat procedure. ( **C** ) Visualization of volcano plot showing DEGs of the microarray datasets. ( **D** ) The Venn diagram showing the distribution of DEGs between individual diseases and their relationship between them. DEGs, differentially expressed genes.

The detailed information of each dataset giving details of the disease condition, sample groups, source of samples, microarray platform used, and references is depicted in Supplementary Table S1 . The dataset GSE17078 23 was selected for VT, and datasets GSE46907, 24 GSE1402, 25 and GSE3365 26 were selected for inflammatory diseases, viz. SLE, RA, and IBD, respectively. The detailed information of each dataset is given in Supplementary Table S1 . The datasets included in this meta-analysis were control/patient studies with a collective number of 27/3, 5/5, 11/26, and 42/85 for VT, SLE, RA, and IBD, respectively. Prior to the common DEG identification between inflammatory diseases and VT, the datasets were preprocessed and normalized using the INMEX tool. It was necessary to reduce the potential study-specific “batch effects,” which was observed using PCA. It was taken care of by the ComBat procedures. Fig. 2A visually examines the sample clustering patterns and distribution of the variable prior to applying the batch adjustment algorithm using a density plot. Fig. 2B visualizes the inter-mixing of all studied samples from the datasets after the batch effect correction. This depicts the removal of the confounding factors because of the nonbiological variations and thus reducing the potential study-specific “batch effects.”

### Identification of Shared Transcriptomics Signature of the DEGs among VT and Inflammatory Diseases (SLE, RA, and IBD)

The implementation of ES and REM statistical analysis of INMEX identified a common transcriptional signature shared between VT and inflammatory diseases. A total of 613 DEGs including 229 over-expressed and 384 under-expressed genes were identified under the significance threshold of adjusted *p* -value <0.05. Fig. 2C illustrates the volcano plot of significant genes which are over-expressed and under-expressed. Based on the values of CombinedES, Cathepsin L (CTSL), C-X-C motif chemokine 3 (CXCL3), C-X-C motif chemokine 5(CXCL5), protein sprouty homolog 2 (SPRY2), transmembrane protein 158 (TMEM158), C-X-C motif chemokine 2 (CXCL2), spermine oxidase (SMOX), a disintegrin and metalloproteinase with thrombospondin motifs 2 (ADAMTS2), epiregulin (EREG), C-X-C motif chemokine ligand 8 (CXCL8), or IL-8 were among the most overexpressed genes with significant *p* -values. While, Ras association domain-containing protein 1 (RASSF1), microtubule-actin cross-linking factor 1 (MACF1), zinc finger and BTB domain-containing protein 16 (ZBTB16), O-linked *N* -acetylglucosamine (GlcNAc) transferase (OGT), gamma-secretase-activating protein (GSAP), E74-like ETS transcription factor 2 (ELF2), nuclear factor of activated T cells 2 interacting protein (NFATC2IP), PNN interacting serine and arginine rich protein (PNISR), phosphorylase kinase catalytic subunit gamma 2 (PHKG2), and E3 ubiquitin-protein ligase (MYCBP2) were the most under-expressed genes with significant *p* -values across the four microarray datasets. The compiled list of significantly over-expressed and under-expressed DEGs from the meta-analysis with combined ES and adjusted *p* -value <0.05 is provided in Supplementary Table S2 . The Venn diagram of significant DEGs depicted in Fig. 2D illustrates the share of significant genes in independent studies and in total as 416 genes lie in VT, 32 genes in SLE, 795 genes in RA, and 3,223 genes in IBD along with 613 DEGs are shared by all the four diseases.

### Chromatin-Modifying Pathway Enrichment

**Fig. 3:**
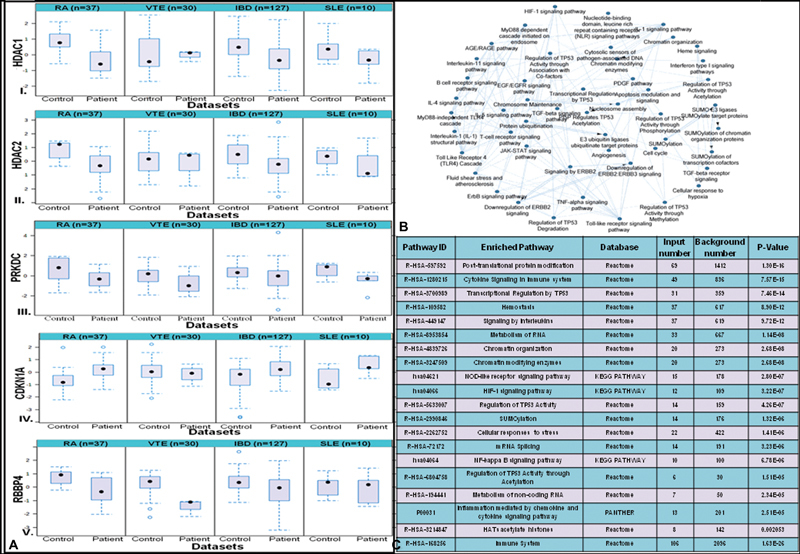
Hub gene expression and their regulated pathways. ( **A** ) Expression pattern of selected hub gene which shows the expression in different disease conditions. I. HDAC1 ( *p* -value = 0.0125), II. HDAC2 ( *p* -value = 0.002), III. PRKDC ( *p* -value = 0.002), IV. CDKN1A ( *p* -value = 0.000769), V. RBBP4 ( *p* -value = 0.0074). ( **B** ) Pathway enrichment interaction showing the significant pathway. Network overrepresentations of enriched pathway and gene ontology integrating the Kyoto Encyclopedia of Genes and Genomes (KEGG) and Reactome pathways for the top 20 hub genes using Cytoscape plug-in, ClueGO. Right-sided hypergeometric distribution tests, with an adjusted *p* -value of 0.05, followed by the Bonferroni adjustment based on the highest significance. ( **C** ) Enriched pathway of shared DEGs using online tool Kobas3.0 in a tabular format. HATs, Histone acetyltransferases; HIF, hypoxia inducible factor; NF-κB, Nuclear factor-κB; Kyoto encyclopedia of genes; NOD, nucleotide-binding, oligomerization domain; TP53, tumor protein p53.

Overrepresented biological pathways and GO terms were identified by gene set enrichment analysis. This was executed by Kobas3.0 tool using the complete list of significant over-expressed and under-expressed DEGs. Fig. 3C represents the results for the enriched biological pathways from various pathway analysis libraries like the KEGG, BioCyc, Reactome pathway, and PANTHER with adjusted *p* -value <0.05 using hypergeometric test/Fisher's exact test with the Benjamini–Hochberg FDR correction method. The present meta-analysis showed posttranslational protein modification (R-HSA-597592), cytokine signaling in the immune system (R-HSA-1280215), transcriptional regulation by TP53 (R-HSA-3700989), hemostasis (2.11E-13), signaling by ILs (R-HSA-449147), chromatin organization (R-HSA-4839726), chromatin-modifying enzymes (R-HSA-3247509), HATs acetylate histones (R-HSA-3214847), transcription regulation by TP53 (R-HSA-3700989), and posttranslational protein modification (R-HSA-597592) are the top enriched significant pathways that regulate the epigenetic control of gene expression as top enriched pathways with corrected *p* -value <0.05.

### Histone-Modifying Enzymes Are among the Top Identified Hub Genes

A PPI network was generated through NetworkAnalyst/INMEX by integrating the String database for the complete list of 609 DEGs. An original PPI network having 2,687 nodes with 5,291 edges was generated; however, for better visualizations of the PPI network, the “minimum interaction network” with 760 nodes showing interaction with 2,144 edges was selected. Based on their topological parameters, viz. degree and betweenness centrality, the key hub genes were extracted using Cytoscape through the network analyzer. Based upon the network topology, the most highly ranked nodes across the four datasets were histone deacetylase 1 (HDAC1; degree = 89, betweenness centrality = 3.91) and histone deacetylase 2 (HDAC2; degree = 62, betweenness centrality = 0.0) followed by Fos Proto-Oncogene (FOS; degree = 46, betweenness centrality = 1.72), protein kinase, DNA-activated, catalytic subunit (PRKDC; degree =41, betweenness centrality =1.74), cyclin-dependent kinase inhibitor 1A (CDKN1A; degree = 36, betweenness centrality = 0.99275), RB binding protein 4, chromatin remodeling factor (RBBP4; degree = 34 betweenness centrality = 0.70), and Erb-B2 receptor tyrosine kinase 2 (ERBB2; degree = 33, betweenness centrality = 1.29). HDAC1 and HDAC2 are the top hub genes with the highest degree which are histone-modifying enzymes. Table 1 shows a list of the top 10 hub genes based on the degree and Fig. 3A illustrates the expression pattern of the top 5 hub genes in all the datasets concerning disease and control. The top pathway enrichment using the ClueGO, a Cytoscape plug-in of the PPI network based on the top 50 hub genes, is shown in Fig. 3C whereas Fig. 3B depicts pathway enrichment in network form where the significant pathways are highlighted.

**Table 1:** List of top 10 hub gene based on their topological parameter such as degree and *p* -value <0.05

Entrez ID	Gene symbol	Degree	Betweenness centrality	Closeness centrality	Combined ES	*p* -Value
3065	HDAC1	89	3.918389	0.666667	−0.80456	0.012591
3066	HDAC2	62	0	0	−0.80045	0.002007
2353	FOS	46	1.721673	0.459854	0.68654	0.042176
5591	PRKDC	41	1.743195	0.440895	−0.6355	0.002753
1026	CDKN1A	36	0.9275	0.392701	0.80497	0.000769
5928	RBBP4	34	0.708424	0.459459	−0.79299	0.007486
2064	ERBB2	33	1.291217	0.36413	−0.5804	0.007874
7528	YY1	33	0.834459	0.370739	−0.56943	0.00908
7329	UBE2I	32	1.077831	0.45	−0.9885	0.001474
1386	ATF2	28	0.833348	0.33694	−0.52418	0.019178

Abbreviations: ATF2, activating transcription factor 2; CDKN1A, cyclin-dependent kinase inhibitor 1A; ERBB2,Erb-B2 receptor tyrosine kinase 2; FOS, Fos proto-oncogene; HDAC1, histone deacetylase 1; HDAC2, histone deacetylase 2; PRKDC, protein kinase, DNA-activated, catalytic subunit; RBBP4, RB binding protein 4, chromatin remodeling factor; UBE2I, ubiquitin conjugating enzyme E2 I; YY1,Yin Yang 1.

### miRNA Interacting Partners of Top 10 Hub Genes

With chromatin-modifying pathway enrichment and histone-modifying enzymes among the top identified hub genes, we were intrigued to screen the epitranscriptomic signature among the shared hub genes. We enlisted miRNA-interacting partners of the top 10 hub genes from our analysis. Supplementary Table S3 shows the interaction of the top 10 hub genes with their miRNA-interacting partners. CDKN1A interacts with 331 miRNAs, FOS interacts with 58 miRNAs, HDAC1 interacts with 10 miRNAs, HDAC2 interacts with 6 miRNAs, and PRKDC interacts with 2 miRNAs.

### Processing and Screening of DEmiRs

In addition to the list of miRNA-interacting partners of the top 10 hub genes, our study also screened shared DEmiR signatures among inflammatory diseases (SLE, RA, and IBD) and VT. For these, miRNA expression datasets were also mined on NCBI GEO for each disease as described earlier ( Fig. 1A ). In total, 7 hits for VT, 27 hits for SLE, 52 hits for RA, and 163 for IBD were observed in the beginning. Filtering the initial results based on organism and study type gave us the remaining 7 hits for VT, 22 hits for SLE, 42 hits for RA, and 138 hits for IBD. These results were then filtered on the basis of entry type and 4 articles for VT, 15 articles for SLE, 37 articles for RA, and 43 articles for IBD were left. These results were again filtered on the basis of title or abstract duplication and observed 1 article for VT, 8 articles for SLE, 16 articles for RA, and 19 articles for IBD. Finally, we selected the datasets with healthy/patient studies, blood, and its component as a source and with a comparable condition from the remaining articles. One miRNA expression dataset for each disease was selected for performing the meta-analysis. A total of four microRNA studies met the inclusion criteria and were selected further for the meta-analysis. The dataset GSE24149 27 was selected for pulmonary embolism (PE), and datasets GSE79240, 28 GSE124373, 29 and GSE32273 30 were selected for inflammatory diseases, viz. SLE, RA, and IBD, respectively. The detailed information of each dataset is given in Supplementary Table S1 . The datasets included in this meta-analysis were control/patient studies with a collective number of 10/10, 5/5, 18/28, and 66/66, for PE, SLE, RA, and IBD, respectively.

**Fig. 4:**
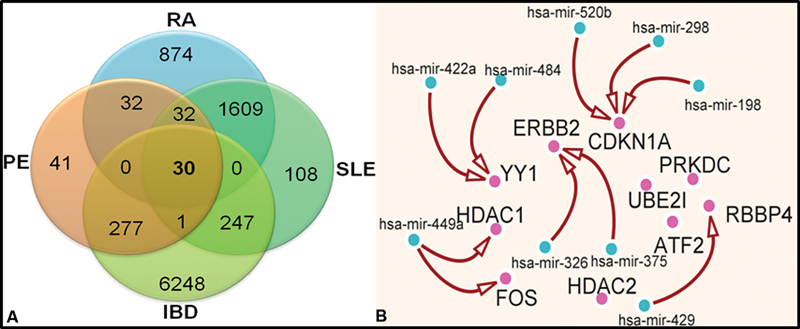
Analysis of miRNA dataset for the identification of DEmiRs and its interaction with the associated DEGs. ( **A** ) The Venn diagram showing the distribution of DEmiRs between individual diseases, their relationship between them, and the common DEmiRs among all the diseases. ( **B** ) Interaction of top 10 hub genes with the common DEmiRs from the microRNA list of the top 10 hub genes and DEmiRs selected from the microRNAs datasets.

After the preprocessing of each miRNA dataset independently, DEmiRs were screened using a *p* -value of less than 0.05 as the threshold. We obtained 376, 2,006, 2,578, and 6,804 DEmiRs from PE, SLE, RA, and IBD, respectively. After this, each dataset was compared with other datasets and in a combinatorial method to find the common DEmiRs with the help of online Bioinformatics and Research computing website tools provided by MIT ( http://barc.wi.mit.edu/tools/compare/index.php ). By comparing each miR dataset in the combinatorial method, only 30 miRNAs were found that were common to all four selected studies. Out of these 30 miRNAs, 23 DEmiRs were up-regulated and 7 DEmiRs were down-regulated. The complete list of DEmiRs from the analysis is provided in Supplementary Table S4 . Some of the up-regulated DEmiRs are hsa-mir-107, hsa-mir-133b, hsa-mir-137, hsa-mir-346, hsa-mir-147b, hsa-mir-198, hsa-mir-301b, hsa-mir-326, and hsa-mir-422a, and some of the down-regulated DEmiRs are hsa-mir-184, hsa-mir-298, and hsa-mir-325. The Venn diagram of significant DEmiRs depicted in Fig. 4A shows the share of significant miRs in independent studies with only 30 DEmiRs that were shared by all four diseases ( Supplementary Table S4 ).

### Common DEmiRs Selected from the list of miRNA-Interacting Partners of Top 10 Hub Genes and Shared Significant DEmiRs from microRNA Dataset Acquisition

The obtained 30 DEmiRs from the independent miRNAs array studies were subjected to comparison with miRNAs obtained from gene–microRNAs interaction on the basis of the top 10 hub genes. A list of 9 DEmiRs was common from the list of miRNA-interacting partners of the top 10 hub genes and the shared 30 DEmiRs from the microRNAs meta-analysis ( Table 2 ). Fig. 4B depicts the interaction of common 9 DEmiRs with their partner DEGs, which are 10 hub genes in the present case. MicroRNAs, has-mir-198, hsa-mir-298, and hsa-mir-520b from the common nine DEmiRs are involved with the CDKN1A gene. MiRNAs, hsa-mir-422a, and hsa-mir-484 play role in regulating the YY1 gene. miRNA hsa-mir-449 regulates HDAC1 and FOS gene, while hsa-mir-429 regulates RBBP4. Finally, miRNAs, hsa-mir-326, and hsa-mir-375 regulate the function of the ERBB2 gene. Thus, a total of six hub genes are demonstrated as the interacting partners for the common nine DEmiRs.

**Table 2:** Total of nine miRNAs that are shared between the two studies' analysis, Hub gene–miRNA interacting partners and miRNA meta-analysis for four datasets, VT/PE, SLE, RA, and IBD with their functional role

Common miRNAs	Their hub genes	Functional role of hub gene	References
hsa-mir-449a	HDAC1, FOS	HDAC1 catalyzes the deacetylation of lysine residues on the N-terminal part of core histones. It serves as an epigenetic repression and modulates transcription. FOS gene is member of genes known as immediate early genes. It heterodimerized with JUN family genes to form activator protein-1 (AP-1) and binds with TGAC/GTCA consensus sequences in the promoter region of target genes.	65 66
hsa-mir-198	CDKN1A	CDKN1A is essential in cellular response to DNA damage and its over-expression due to p53 checkpoint pathway leads to cell arrest.	67
hsa-mir-298			
hsa-mir-520b			
hsa-mir-429	RBBP4	RBBP4 exists in protein complexes and plays a significant role in histone acetylation, methylation, and chromatin-modifying complexes.	68
hsa-mir-326	ERBB2	ERBB2 is present in protein complex involved in chromatin remodeling and modulates transcription with histone acetylation/deacetylation.	69
hsa-mir-375			
hsa-mir-422a	YY1	YY1 is a transcription factor that modulates transcription. It regulates histone alpha complex and replication.	70
hsa-mir-484			

Abbreviations: AP-1, activator protein 1; CDKN1A, cyclin-dependent kinase inhibitor 1A; ERBB2,Erb-B2 receptor tyrosine kinase 2; FOS, Fos, proto-oncogene; HDAC1, histone deacetylase 1; RBBP4, RB binding protein 4, chromatin remodeling factor; TP53, tumor protein p53; YY1,Yin Yang 1.

## Discussion

A diseased condition that causes inflammation may also alter the hemostatic balance leading to endothelial dysfunction and activation, platelet and immune cell activation, and the release of various cytokines. All these alterations create a prothrombotic milieu in several inflammatory diseases like SLE, RA, and IBD. 8 9 10 11 12 13 However, the underlying molecular mechanisms of the thrombotic events are still largely obscure. Microarray studies have enabled the acquisition of large quantities of data for specific settings, but the small sample size of these studies remains a major limiting factor. Performing a meta-analysis of multiple available microarray datasets not only increases the sample size but also provides the opportunity to identify shared DEGs with greater confidence and authenticity. Thus, the present work attempts to identify shared gene signatures between inflammatory pathology observed in inflammatory diseases such as SLE, RA, and IBD with VT. 31 32 33 34 35 36 37 Publicly available microarray datasets for VT, SLE, RA, and IBD identified the shared transcriptomics and epitranscriptomics signatures among them. The meta-analysis provided 613 shared DEGs including 229 overexpressed and 384 under-expressed genes under the significance threshold of adjusted *p* -value <0.05. The functions of shared DEGs were ascertained through pathway enrichment analysis and hub gene identification using the comprehensive enrichment library of Kobas3.0 and Cytoscape. Chromatin-modifying pathway, histone-modifying enzymes, hypoxia-related pathways, activation of “cytokine signaling in immune system,” “inflammation mediated by chemokine and cytokine signaling pathway,” “metabolism of RNA,” “transcriptional regulation,” and “platelet activation” were among the enriched biological pathways. All of these enriched pathways indicated hypoxia-related pathways along with chromatin-modifying pathways such as chromatin organization, chromatin-modifying enzymes, HAT acetylate histones, and histone-modifying enzymes. Hypoxia-related pathways show a strong correlation with inflammation and coagulation through an interplay between HIF1α and inflammasome. 3 Likewise, chromatin-modifying pathways such as chromatin-modifying enzymes, HAT acetylate histones, and histone-modifying enzymes suggest the influence of epigenetic changes on the occurrence and progression of thrombotic diseases. 38 For example, histones play a significant role in the manifestation of thrombosis and inflammation. 38 Histone H4 binds to prothrombin and by autoactivation generates thrombin. 39 Histone also accelerates fibrin formation and induces platelet activation in a Toll-like receptors-2 (TLRs-2) and TLR-4-dependent manner. 40 41 Histones can bind to protein C and thrombomodulin and impair protein C activation. 42 Further, histone complexes with DNA can impair fibrinolysis by binding to fibrin. 43 The procoagulant effects of histones, coupled with their ability to kill endothelial cells, activate platelets and inhibit fibrinolysis, contribute to tissue injury and microvascular thrombosis. Evidence of epigenetic regulators in our mRNA meta-analysis intrigued us to look for epitranscriptomics profiles targeting miRNAs in the mentioned diseased conditions as well. Human miRNAs like miR-126 and miR-146a regulate the expression of genes involved in the pathways leading to immunothrombosis. 44 45 Further, the altered biogenesis of miRNAs like miR-15, miR-125a, miR-142, miR-146a, miR-155, and miR-181 is observed in patients suffering from SLE. 46 Another study on SLE showed a rise in the expression of pro-inflammatory miRNAs and a decline in anti-inflammatory miR146a in the peripheral blood mononuclear cell (PBMCs) isolated from patient subjects as compared to healthy subjects. 47 Similarly, miR-21 and miR-124 are found to increase inflammation leading to IBD, while miR-146a and miR-155 are altered in RA. 48 49 Sahu et al have demonstrated that a decrease in the expression of miR-145 in PBMCs, platelets, vascular endothelial, and smooth muscle cells is associated with the development of thrombus. 50 Restoration of normal miR-145 levels in thrombotic animals further resulted in reduced thrombogenesis via decreased tissue factor levels. 50 Thus, examining shared miRNA involved in the interplay of inflammation and thrombosis is crucial. A total of 30 miRNAs with adjusted *p* -value <0.05 were identified as a shared DEmiRs signature among inflammatory diseases (SLE, RA, and IBD) and VT. These 30 DEmiRs were subjected to comparison with miRNAs obtained from gene–microRNAs interaction of the top 10 hub genes that gave nine miRNAs. The miRNAs such as has-mir-449a targets HDAC1 and FOS, hsa-mir-198, hsa-mir-298, and has-mir-520b target CDKN1A, hsa-mir-429 targets RBBP4, hsa-mir-326 and hsa-mir-375 target ERBB2, and hsa-mir-422a and hsa-mir-484 target YY1. hsa-mir-449a regulates HDAC1 and FOS genes, which plays a significant role in posttranslational protein modification, transcriptional regulation by TP53, hemostasis, chromatin organization, chromatin-modifying enzymes, and SUMOylation. 51 52 Studies have demonstrated downregulation of endothelial nitric oxide synthase (eNOS) expression during the silencing of HDAC1 by hsa-mir-449a, which indicates their involvement in maintaining the integrity of endothelial cells whose functions are pivotal in inflammatory and coagulatory responses. 53 Hsa-mir-198, has-mir-298, and hsa-mir-520b modulate the expression of CDKN1A, which is involved in cytokine signaling in the immune system, signaling by IL, HIF-1 signaling pathway, and cellular response to stress. 54 Hsa-mir-326 and hsa-mir-375 regulate ERBB2, which is involved in pathways like signaling by IL and HIF. 55 56 Over-expression of ERBB2 is associated with inflammation in disease pathologies. 57 Hsa-mir-422a and hsa-mir-484 modulate the function of YY1 gene associated with posttranslational protein modification to regulate inflammation through different pathways like TLRs, NOD-like receptors, and inflammasome. 58 Additionally, Hsa-mir-484 is reported in endothelial dysfunction by alleviating the expression of eNOS 59 and has been involved in coronary artery disease, cardiac ischemia-related diseases, and inflammation including others. 60 Our results indicate a strong association of epigenetic changes and histone modification with miRNAs in inflammation and coagulation and gave us an insight into the involvement of epigenetic pathways in the context of immunothrombosis and inflammatory disease. The study gave us specific pathways and their interconnections with pathological miRNA that exhibited a significant causal influence on driving inflammation-related thrombosis. However, there are certain limitations to the study that should be discussed. All of the publicly available microarray datasets used in the studies are derived from blood cells such as PBMCs that may not be an ideal model to understand tissue or organ-specific inflammatory or thrombotic responses; however, PBMCs can mirror inflammatory changes within the body 61 62 and can enable the real-time monitoring of the patients and their disease progression and responses of the treatment. In addition, dataset-specific information from the individual microarray dataset could get lost when integrating multiple microarray datasets using meta-analysis. Integration unintentionally adds confounding factors that mask the real biological signals, inadvertently introduce or amplify biases, and produce erroneous relationships. However, the usage of a random effect model, as is the case in this study, can help in minimizing the biases if not circumventing them. A random effect model employs different weights to different studies such that smaller studies are often given less weight in the analysis than larger studies with more participants. Hence, it assists in controlling unobserved heterogeneity. Furthermore, the ComBat procedure to remove the batch effect can excessively influence the data and inadvertently mask true biological variation. 63 64 ComBat procedures make certain assumptions about the data distribution that could compromise its functionality and may increase the chance of overfitting when there are few samples in a batch. Although there are previous individual gene expression microarray reports on either individual diseases or in combination, the present work, however, is the first one, as per our knowledge, where datasets of mRNA and miRNAs on these four disorders have been integrated, to define shared biological processes. In addition, the work provides biological insights into the mechanisms underlying the modulation of the coagulation cascade.
