# Supplementary material for: Unraveling Epigenetic Interplay between Inflammation, Thrombosis, and Immune-Related Disorders through a Network Meta-analysis
Source: TH Open. 2024 Feb 2;8(1):e81–92. doi: 10.1055/a-2222-9126 (PMC10837039; doi:10.1055/a-2222-9126)
Supplement: Supplementary file 1 — Supplementary Material [file 10-1055-a-2222-9126-s23070028.pdf]

Supplementary Data

Supplementary Table S1 Depiction of the details of all the datasets used for the meta-analysis with the details of sample size, source of sample, platform used for analysis and references

GEO Accession NO.	Disease	Samples(Control/Patient)	Source of Sample	Platform	Reference
GSE17078	Venous Thrombosis	(N = 30) 27/3	BOECs	Affymetrix Human Genome U133A 2.0 Array	23
GSE24149	Acute Pulmonary Embolism	(N = 20) 10/10	Plasma	Tongji University School of Medicine TaqMan MicroRNA Array v2.0 A	27
GSE46907	Systemic lupus erythematosus	(N = 10) 5/5	Blood Monocytes	Affymetrix Human Genome U133A Array	24
GSE79240	Systemic lupus erythematosus	(N = 10) 5/5	Dendritic cells	Agilent Unrestricted Human miRNA-v19 microarray	28
GSE1402	Rheumatoid arthritis	(N = 37) 11/26	PBMCs	Affymetrix Human Genome U95A 2.0 Array	25
GSE124373	Rheumatoid arthritis	(N = 46) 18/28	PBMCs	Affymetrix Multispecies miRNA-4 Array	29
GSE3365	Inflammatory Bowel Syndrome	(N = 127) 42/85	PBMCs	Affymetrix Human Genome U133A Array	26
GSE32273	Inflammatory Bowel Syndrome (Ulcerative Colitis)	(N = 132)66/66	Blood (Micro- vesicles, PBMCs, Platelets)	Affymetrix Multispecies miRNA-1 Array	30

Abbreviations: BOEC, blood outgrowth endothelial cells; PBMCs, peripheral blood mononuclear cells

 © 2024. The Author(s).

Supplementary Table S2 The complete list of differentially expressed genes (DEGs) with combinedES and p values

EntrezID	Name	CombinedES	Pval
1514	CTSL	1.9837	0.012319
2921	CXCL3	1.5887	0.011271
6374	CXCL5	1.4735	0.036071
10253	SPRY2	1.3034	0.003166
25907	TMEM158	1.2349	0.013838
2920	CXCL2	1.2316	5.65E-10
54498	SMOX	1.2172	0.028055
9509	ADAMTS2	1.2157	0.04951
2069	EREG	1.2005	0.009163
3576	CXCL8	1.1779	2.26E-09
22885	ABLIM3	1.1768	0.007844
58528	RRAGD	1.165	3.50E-09
5197	PF4V1	1.1577	4.49E-09
7850	IL1R2	1.1403	6.64E-09
2752	GLUL	1.1258	0.000581
4354	MPP1	1.1254	1.19E-08
7076	TIMP1	1.1046	1.48E-08
22822	PHLDA1	1.1021	1.83E-08
5329	PLAUR	1.1017	0.001715
9021	SOCS3	1.0697	4.63E-08
5627	PROS1	1.0689	0.000191
5046	PCSK6	1.054	0.024797
8969	H2AC11	1.0512	0.014248
2769	GNA15	1.0435	0.009858
2180	ACSL1	1.0409	2.36E-05
55353	LAPTM4B	1.0329	0.00354
5912	RAP2B	1.0276	3.78E-05
7171	TPM4	1.0192	2.71E-07
8061	FOSL1	0.99296	0.008048
3638	INSIG1	0.99164	5.34E-07
6515	SLC2A3	0.98967	5.34E-07
6354	CCL7	0.98759	0.000717
10221	TRIB1	0.98596	0.002723
2354	FOSB	0.98235	8.09E-07
3934	LCN2	0.9799	8.25E-07
967	CD63	0.97111	0.000159
3142	HLX	0.97027	8.97E-07
8970	H2BC11	0.96604	0.037553
5476	CTSA	0.96389	0.002386
3690	ITGB3	0.96057	7.34E-05
7504	XK	0.94741	0.010735
597	BCL2A1	0.94122	0.012639
212	ALAS2	0.94077	2.33E-06

 © 2024. The Author(s).

Supplementary Table S2 (*Continued*)

EntrezID	Name	CombinedES	Pval
1749	DLX5	0.92969	0.007912
27030	MLH3	0.92904	0.001859
4783	NFIL3	0.92693	0.003892
5154	PDGFA	0.92613	3.46E-06
6383	SDC2	0.92532	0.038602
10105	PPIF	0.91729	3.99E-06
3586	IL10	0.91592	1.46E-05
6344	SCTR	0.89578	0.005086
6446	SGK1	0.88327	0.000801
3761	KCNJ4	0.87676	0.009094
136	ADORA2B	0.86361	1.53E-05
6385	SDC4	0.8631	8.39E-05
1950	EGF	0.86305	1.52E-05
3953	LEPR	0.85665	1.93E-05
3914	LAMB3	0.85421	1.93E-05
8344	H2BC6	0.85157	0.048291
54741	LEPROT	0.85127	2.13E-05
7378	UPP1	0.84713	0.006646
1847	DUSP5	0.84524	0.031539
5328	PLAU	0.84426	0.03008
7512	XPNPEP2	0.8416	2.57E-05
8347	H2BC4	0.83935	2.93E-05
395	ARHGAP6	0.82964	3.80E-05
4860	PNP	0.82458	0.014248
1910	EDNRB	0.81936	4.42E-05
11343	MGLL	0.81799	0.013718
10661	KLF1	0.81558	4.83E-05
1293	COL6A3	0.81479	0.022886
1259	CNGA1	0.8066	6.82E-05
1026	CDKN1A	0.80497	0.000769
5625	PRODH	0.8028	0.000262
7130	TNFAIP6	0.80107	0.046391
123	PLIN2	0.79952	0.015648
5045	FURIN	0.79926	7.89E-05
3044	HBBP1	0.79534	8.39E-05
7111	TMOD1	0.78605	0.000103
7421	VDR	0.78574	0.000106
6541	SLC7A1	0.78519	0.000111
7277	TUBA4A	0.785	0.042001
7077	TIMP2	0.77426	0.021336
3671	ISLR	0.7731	0.005473
9625	AATK	0.76878	0.048231
6521	SLC4A1	0.76491	0.011398

(*Continued*)

 © 2024. The Author(s).

Supplementary Table S2 (*Continued*)

EntrezID	Name	CombinedES	Pval
5138	PDE2A	0.76328	0.000178
1604	CD55	0.7592	0.000191
7533	YWHAH	0.75782	0.008241
9516	LITAF	0.75088	0.000241
9023	CH25H	0.74982	0.000232
27340	UTP20	0.74849	0.035824
4149	MAX	0.74733	0.000263
717	C2	0.74682	0.048227
1687	GSDME	0.74049	0.000294
3012	H2AC8	0.72594	0.000395
6855	SYP	0.71156	0.000581
3569	IL6	0.70908	0.00063
2273	FHL1	0.70539	0.000683
8612	PLPP2	0.70099	0.000728
9058	SLC13A2	0.70021	0.000728
7035	TFPI	0.69715	0.002586
3500	IGHG1	0.69604	0.000804
6016	RIT1	0.69313	0.000827
27296	TP53TG5	0.69122	0.000869
2353	FOS	0.68654	0.042176
759	CA1	0.68436	0.008052
27102	EIF2AK1	0.68225	0.001101
10211	FLOT1	0.68014	0.037735
1508	CTSB	0.67937	0.01756
23650	TRIM29	0.67548	0.029531
23071	ERP44	0.66585	0.001526
2303	FOXC2	0.66431	0.001557
4311	MME	0.65852	0.001831
4133	MAP2	0.65708	0.001855
10912	GADD45G	0.65672	0.00215
29957	SLC25A24	0.65605	0.00791
7320	UBE2B	0.6514	0.002096
5141	PDE4A	0.64559	0.007844
7323	UBE2D3	0.64499	0.002321
286	ANK1	0.64397	0.002314
200734	SPRED2	0.64143	0.004562
6611	SMS	0.63964	0.007764
10867	TSPAN9	0.63943	0.002696
1326	MAP3K8	0.63294	0.003029
1471	CST3	0.62878	0.003091
1775	DNASE1L2	0.62853	0.003162
23660	ZKSCAN5	0.62748	0.00323
4342	MOS	0.62405	0.034528

 © 2024. The Author(s).

Supplementary Table S2 (*Continued*)

EntrezID	Name	CombinedES	Pval
634	CEACAM1	0.62259	0.003495
1413	CRYBA4	0.62035	0.032396
2520	GAST	0.62002	0.003742
5872	RAB13	0.61986	0.003795
8613	PLPP3	0.61918	0.003788
2152	F3	0.61401	0.004015
7429	VIL1	0.61359	0.029471
114548	NLRP3	0.61304	0.004286
7803	PTP4A1	0.61279	0.004108
8754	ADAM9	0.60831	0.038574
9957	HS3ST1	0.60739	0.004727
5626	PROP1	0.60445	0.004853
9536	PTGES	0.6039	0.004977
2258	FGF13	0.60195	0.005162
9717	SEC14L5	0.60133	0.005252
3149	HMGB3	0.60045	0.005309
9473	THEMIS2	0.60016	0.015775
3764	KCNJ8	0.5988	0.005473
3340	NDST1	0.59243	0.006058
24	ABCA4	0.59031	0.048715
51560	RAB6B	0.58741	0.033903
26227	PHGDH	0.58672	0.006712
9640	ZNF592	0.58653	0.006815
3514	IGKC	0.58533	0.007001
10882	C1QL1	0.58071	0.007687
11332	ACOT7	0.58021	0.00760
598	BCL2L1	0.57892	0.008002
7280	TUBB2A	0.57866	0.007844
2840	GPR17	0.57624	0.008021
4780	NFE2L2	0.57384	0.008497
201191	SAMD14	0.57378	0.008535
25891	PAMR1	0.57366	0.008432
189	AGXT	0.57356	0.008497
2696	GIPR	0.57072	0.008665
4084	MXD1	0.56856	0.009094
2335	FN1	0.56676	0.009378
4664	NAB1	0.56212	0.04449
3215	HOXB5	0.56073	0.010441
8993	PGLYRP1	0.55878	0.011081
7805	LAPTM5	0.55739	0.011398
2935	GSPT1	0.55558	0.019976
3162	HMOX1	0.55419	0.011646
5306	PITPNA	0.55065	0.012591

(*Continued*)

 © 2024. The Author(s).

Supplementary Table S2 (*Continued*)

EntrezID	Name	CombinedES	Pval
2184	FAH	0.55056	0.012238
4956	ODF1	0.55001	0.012603
26037	SIPA1L1	0.54891	0.013173
1813	DRD2	0.54792	0.029467
80852	GRIP2	0.5479	0.01324
1804	DPP6	0.5464	0.013675
6752	SSTR2	0.54601	0.013767
1261	CNGA3	0.54474	0.013675
4636	MYL5	0.54398	0.013838
1351	COX8A	0.54391	0.013675
23210	JMJD6	0.54116	0.014332
55565	ZNF821	0.53838	0.015328
22904	SBNO2	0.53768	0.015328
176	ACAN	0.53723	0.01561
771	CA12	0.53649	0.015331
3248	HPGD	0.53413	0.016452
8555	CDC14B	0.53042	0.017133
5444	PON1	0.52948	0.017654
26608	TBL2	0.52916	0.017839
6533	SLC6A6	0.52787	0.017983
23764	MAFF	0.52673	0.018478
2239	GPC4	0.52646	0.017915
3680	ITGA9	0.5242	0.01864
9563	H6PD	0.52381	0.019549
1746	DLX2	0.52318	0.019464
439921	MXRA7	0.5202	0.02049
5968	REG1B	0.51814	0.020898
5156	PDGFRA	0.51723	0.021202
5025	P2RX4	0.51586	0.02179
7434	VIPR2	0.51519	0.022089
571	BACH1	0.51358	0.022303
2171	FABP5	0.50874	0.024493
9127	P2RX6	0.50373	0.026976
1805	DPT	0.50224	0.027227
9322	TRIP10	0.5005	0.02788
3678	ITGA5	0.49909	0.029665
26330	GAPDHS	0.4982	0.029869
4616	GADD45B	0.4973	0.035066
1158	CKM	0.4951	0.04626
2996	GYPE	0.48977	0.033903
5949	RBP3	0.48883	0.034871
3720	JARID2	0.48881	0.034528
881	CCIN	0.4886	0.033903

 © 2024. The Author(s).

Supplementary Table S2 (*Continued*)

EntrezID	Name	CombinedES	Pval
51375	SNX7	0.48824	0.034871
6820	SULT2B1	0.48666	0.035612
8605	PLA2G4C	0.483	0.037353
65012	SLC26A10	0.48094	0.038161
2001	ELF5	0.4805	0.039018
6623	SNCG	0.48022	0.038782
51304	ZDHHC3	0.47641	0.042001
8326	FZD9	0.47625	0.042001
479	ATP12A	0.47411	0.042001
420	ART4	0.47058	0.045972
25999	CLIP3	0.46801	0.047361
10381	TUBB3	0.46789	0.047523
7416	VDAC1	0.46593	0.047852
2139	EYA2	0.46447	0.049119
11186	RASSF1	-1.3294	7.14E-11
23499	MACF1	-1.3171	0.001472
7704	ZBTB16	-1.3041	9.71E-11
8473	OGT	-1.2913	9.71E-11
54103	GSAP	-1.2831	1.36E-10
1998	ELF2	-1.235	0.009094
84901	NFATC2IP	-1.2333	5.65E-10
25957	PNISR	-1.2096	9.40E-10
5261	PHKG2	-1.1594	0.013675
23077	MYCBP2	-1.1584	4.49E-09
8880	FUBP1	-1.1581	6.69E-08
6601	SMARCC2	-1.1559	4.49E-09
10219	KLRG1	-1.131	8.74E-09
1854	DUT	-1.1265	0.000135
80342	TRAF3IP3	-1.1211	1.19E-08
56948	SDR39U1	-1.1119	1.44E-08
64764	CREB3L2	-1.0836	3.37E-08
10987	COPS5	-1.0714	5.03E-08
23613	ZMYND8	-1.0629	0.027341
50853	VILL	-1.0519	0.007911
4297	KMT2A	-1.045	0.015331
27352	SGSM3	-1.0377	0.006193
51747	LUC7L3	-1.0355	1.66E-07
22861	NLRP1	-1.0347	0.000764
22914	KLRK1	-1.0307	2.31E-05
1459	CSNK2A2	-1.0237	0.025209
9736	USP34	-1.021	0.010505
8558	CDK10	-1.0118	3.17E-07
8019	BRD3	-1.0102	3.17E-07

(*Continued*)

 © 2024. The Author(s).

Supplementary Table S2 (*Continued*)

EntrezID	Name	CombinedES	Pval
5525	PPP2R5A	-0.99786	0.020007
8192	CLPP	-0.99739	0.006712
7329	UBE2I	-0.9885	0.001474
10198	MPHOSPH9	-0.98612	1.34E-05
2734	GLG1	-0.98245	0.03251
51433	ANAPC5	-0.982	8.09E-07
1120	CHKB	-0.9775	0.000454
58487	CREBZF	-0.97499	8.75E-07
3631	INPP4A	-0.97024	1.01E-06
10180	RBM6	-0.96974	9.42E-07
8165	AKAP1	-0.96232	1.18E-06
26999	CYFIP2	-0.96178	1.23E-06
9924	PAN2	-0.95975	1.34E-06
9057	SLC7A6	-0.9486	1.64E-06
167227	DCP2	-0.94777	1.64E-06
22906	TRAK1	-0.94725	0.007819
6599	SMARCC1	-0.94535	0.016452
7593	MZF1	-0.9446	1.78E-06
3824	KLRD1	-0.9444	0.024035
8455	ATRN	-0.93858	0.049119
4775	NFATC3	-0.93577	0.008186
8216	LZTR1	-0.93012	2.66E-06
57125	PLXDC1	-0.93005	6.12E-06
728093	728093	-0.92263	0.001745
546	ATRX	-0.91475	3.99E-06
11083	DIDO1	-0.91383	4.96E-06
83988	NCALD	-0.91317	0.003836
157680	VPS13B	-0.91146	5.10E-06
9354	UBE4A	-0.90858	5.10E-06
9931	HELZ	-0.90768	0.010735
825	CAPN3	-0.90517	5.66E-06
818	CAMK2G	-0.9	6.12E-06
55556	ENOSF1	-0.8974	7.39E-06
6311	ATXN2	-0.89601	0.020498
3004	GZMM	-0.89448	7.87E-06
79026	AHNAK	-0.89414	0.000306
10142	AKAP9	-0.89279	0.039643
9016	SLC25A14	-0.89243	7.87E-06
8893	EIF2B5	-0.88792	0.003051
3590	IL11RA	-0.88635	8.55E-06
643314	KIAA0754	-0.88574	0.008881
9013	TAF1C	-0.88456	8.63E-06
8525	DGKZ	-0.88444	0.020467

 © 2024. The Author(s).

Supplementary Table S2 (*Continued*)

EntrezID	Name	CombinedES	Pval
259197	NCR3	-0.88323	9.44E-06
29894	CPSF1	-0.88275	0.03194
1605	DAG1	-0.87917	0.008535
7862	BRPF1	-0.87667	0.00028
23513	SCRIB	-0.87663	1.23E-05
10531	PITRM1	-0.87495	0.030907
3799	KIF5B	-0.87435	0.016014
26137	ZBTB20	-0.87338	0.003566
6651	SON	-0.87333	1.23E-05
5378	PMS1	-0.8725	0.000265
9110	MTMR4	-0.87128	1.27E-05
56890	MDM1	-0.86637	1.53E-05
57035	RSRP1	-0.86117	4.28E-05
5590	PRKCZ	-0.86037	1.70E-05
10494	STK25	-0.8591	1.70E-05
3978	LIG1	-0.85767	1.87E-05
4329	ALDH6A1	-0.85728	1.83E-05
29799	YPEL1	-0.85678	0.036776
4832	NME3	-0.85509	1.81E-05
10036	CHAF1A	-0.84888	0.006404
6565	SLC15A2	-0.84232	2.60E-05
9667	SAFB2	-0.83949	2.93E-05
22889	KHDC4	-0.83876	0.015063
784	CACNB3	-0.8376	2.68E-05
4603	MYBL1	-0.83525	0.016208
11340	EXOSC8	-0.83087	0.041129
8888	MCM3AP	-0.8306	0.045885
905	CCNT2	-0.83033	0.003763
55251	PCMTD2	-0.82905	3.46E-05
3710	ITPR3	-0.82573	4.17E-05
7267	TTC3	-0.82084	0.032297
23353	SUN1	-0.81995	0.003025
10464	PIBF1	-0.81686	0.001859
10128	LRPPRC	-0.81403	0.015322
2145	EZH1	-0.81389	5.13E-05
55623	THUMPD1	-0.80889	6.29E-05
65110	UPF3A	-0.80887	0.005005
3065	HDAC1	-0.80456	0.012591
57205	ATP10D	-0.80335	7.34E-05
79161	TMEM243	-0.80279	7.31E-05
6742	SSBP1	-0.80098	7.86E-05
3066	HDAC2	-0.80045	0.002007
549	AUH	-0.7978	0.014709

(*Continued*)

 © 2024. The Author(s).

Supplementary Table S2 (*Continued*)

EntrezID	Name	CombinedES	Pval
8906	AP1G2	-0.7953	8.39E-05
3811	KIR3DL1	-0.79431	8.24E-05
5928	RBBP4	-0.79299	0.007486
51601	LIPT1	-0.79287	0.01679
10150	MBNL2	-0.79181	0.003072
1.01E+08	RPARP-AS1	-0.78984	9.48E-05
23193	GANAB	-0.78754	0.011121
65123	INTS3	-0.7874	0.000103
55000	TUG1	-0.78735	0.000106
9882	TBC1D4	-0.78665	0.00011
6936	GCFC2	-0.78526	0.000581
178	AGL	-0.78363	0.000111
6919	TCEA2	-0.78328	0.015331
54535	CCHCR1	-0.78057	0.000118
6894	TARBP1	-0.78033	0.000116
57794	SUGP1	-0.77955	0.000117
10994	ILVBL	-0.7777	0.000133
3633	INPP5B	-0.77491	0.033903
9894	TELO2	-0.77399	0.000135
25900	IFFO1	-0.77349	0.000141
4869	NPM1	-0.77259	0.000147
27332	ZNF638	-0.77136	0.00015
11278	KLF12	-0.7686	0.000157
23547	LILRA4	-0.76674	0.000159
7433	VIPR1	-0.76596	0.046289
50717	DCAF8	-0.7634	0.000166
5445	PON2	-0.76146	0.000177
6595	SMARCA2	-0.7606	0.00018
23306	NEMP1	-0.7561	0.014798
253959	RALGAPA1	-0.75571	0.000395
10922	FASTK	-0.75498	0.002314
5692	PSMB4	-0.75449	0.000205
10902	BRD8	-0.75108	0.000217
23236	PLCB1	-0.74944	0.000249
84726	PRRC2B	-0.74481	0.000261
55705	IPO9	-0.74478	0.014248
26191	PTPN22	-0.7441	0.000274
11168	PSIP1	-0.74152	0.000274
8543	LMO4	-0.74081	0.000287
7840	ALMS1	-0.73971	0.00028
1385	CREB1	-0.73676	0.000309
5479	PPIB	-0.73459	0.030746
22994	CEP131	-0.73425	0.003742

 © 2024. The Author(s).

Supplementary Table S2 (*Continued*)

EntrezID	Name	CombinedES	Pval
9987	HNRNPDL	-0.7327	0.002586
23598	PATZ1	-0.73233	0.000345
81576	CCDC130	-0.73218	0.01756
3551	IKBKB	-0.73049	0.000345
23234	DNAJC9	-0.72725	0.0004
55644	OSGEP	-0.72426	0.003768
86	ACTL6A	-0.72389	0.040995
54987	CZIB	-0.72349	0.010027
23244	PDS5A	-0.72322	0.001448
9254	CACNA2D2	-0.71863	0.001711
8405	SPOP	-0.7178	0.045885
5607	MAP2K5	-0.71265	0.000537
64410	KLHL25	-0.71154	0.010505
23229	ARHGEF9	-0.71149	0.037693
57634	EP400	-0.71074	0.000581
3550	IK	-0.70989	0.000621
5893	RAD52	-0.7098	0.007519
23461	ABCA5	-0.70797	0.025364
171023	ASXL1	-0.70716	0.000607
7597	ZBTB25	-0.70564	0.000648
6418	SET	-0.705	0.000643
7415	VCP	-0.70274	0.026621
9735	KNTC1	-0.70035	0.024782
27240	SIT1	-0.70033	0.000764
53916	RAB4B	-0.69923	0.000764
7710	ZNF154	-0.69751	0.000764
730092	RRN3P1	-0.69717	0.004004
83637	ZMIZ2	-0.69634	0.000764
2648	KAT2A	-0.69633	0.002136
1936	EEF1D	-0.69487	0.046289
7248	TSC1	-0.6947	0.000804
6433	SFSWAP	-0.69406	0.00082
10299	MARCHF6	-0.69402	0.000847
116984	ARAP2	-0.69389	0.000827
8672	EIF4G3	-0.69321	0.00082
26019	UPF2	-0.69247	0.000869
10919	EHMT2	-0.69199	0.003051
5536	PPP5C	-0.69012	0.000863
1628	DBP	-0.68998	0.000883
7398	USP1	-0.68731	0.018865
4841	NONO	-0.68587	0.00669
22883	CLSTN1	-0.68501	0.000993
594	BCKDHB	-0.6839	0.001047

(*Continued*)

 © 2024. The Author(s).

Supplementary Table S2 (*Continued*)

EntrezID	Name	CombinedES	Pval
25898	RCHY1	-0.68308	0.004192
203	AK1	-0.68289	0.001047
7716	VEZF1	-0.6769	0.001237
5162	PDHB	-0.67557	0.004756
23405	DICER1	-0.67505	0.001225
51499	TRIAP1	-0.67464	0.001317
51594	NBAS	-0.67326	0.002609
1632	ECI1	-0.67225	0.001321
220988	HNRNPA3	-0.67158	0.005224
7109	TRAPPC10	-0.66831	0.001507
1E + 08	1E + 08	-0.66713	0.001448
92249	LINC01278	-0.66665	0.001507
2592	GALT	-0.66613	0.025364
9677	PPIP5K1	-0.66486	0.030907
56941	HMCES	-0.66408	0.002723
25970	SH2B1	-0.65701	0.001801
643641	ZNF862	-0.6564	0.00187
7707	ZNF148	-0.65625	0.001841
1043	CD52	-0.65612	0.037015
10521	DDX17	-0.65511	0.00187
10320	IKZF1	-0.65433	0.049119
26278	SACS	-0.65095	0.002096
285527	FRYL	-0.65027	0.002136
2027	ENO3	-0.64759	0.003768
79090	TRAPPC6A	-0.64532	0.00233
9877	ZC3H11A	-0.64408	0.002321
9326	ZNHIT3	-0.64035	0.002586
5885	RAD21	-0.64025	0.002586
9649	RALGPS1	-0.63934	0.00488
8857	FCGBP	-0.63773	0.002717
26017	FAM32A	-0.63773	0.002723
10013	HDAC6	-0.63724	0.002723
10628	TXNIP	-0.63662	0.019818
26005	C2CD3	-0.63658	0.019721
5591	PRKDC	-0.6355	0.002753
6480	ST6GAL1	-0.63356	0.002904
3595	IL12RB2	-0.63279	0.003025
7019	TFAM	-0.63205	0.003048
23279	NUP160	-0.63043	0.003029
11034	DSTN	-0.62853	0.003162
23294	ANKS1A	-0.62818	0.033308
8481	OFD1	-0.62809	0.03896
924	CD7	-0.62608	0.028483

 © 2024. The Author(s).

Supplementary Table S2 (*Continued*)

EntrezID	Name	CombinedES	Pval
10084	PQBP1	-0.62333	0.015259
10443	N4BP2L2	-0.62244	0.003645
6938	TCF12	-0.61997	0.003742
7443	VRK1	-0.61929	0.003841
4176	MCM7	-0.61857	0.003836
10274	STAG1	-0.61792	0.003768
5203	PFDN4	-0.61779	0.003938
7486	WRN	-0.61697	0.008902
4926	NUMA1	-0.61476	0.032168
5863	RGL2	-0.61427	0.004015
959	CD40LG	-0.61422	0.004177
5709	PSMD3	-0.61397	0.011646
11059	WWP1	-0.61142	0.004492
9039	UBA3	-0.6062	0.004863
3394	IRF8	-0.60487	0.004863
55658	RNF126	-0.6041	0.004992
9855	FARP2	-0.60333	0.005016
8733	GPAA1	-0.60122	0.00522
22900	CARD8	-0.59997	0.005418
5300	PIN1	-0.59986	0.021639
9169	SCAF11	-0.59951	0.005338
10898	CPSF4	-0.59859	0.005391
22937	SCAP	-0.59838	0.005635
57187	THOC2	-0.59718	0.005684
9923	ZBTB40	-0.59567	0.005765
261726	TIPRL	-0.59541	0.005684
203069	R3HCC1	-0.59289	0.023384
2339	FNTA	-0.59209	0.006058
80184	CEP290	-0.59133	0.009822
9743	ARHGAP32	-0.59076	0.006469
5330	PLCB2	-0.58926	0.006685
10236	HNRNPR	-0.58532	0.006898
1112	FOXN3	-0.58437	0.006974
25809	TTLL1	-0.58162	0.007627
2064	ERBB2	-0.5804	0.007874
291	SLC25A4	-0.58001	0.010105
1524	CX3CR1	-0.57902	0.010707
4214	MAP3K1	-0.57897	0.00791
64795	RMND5A	-0.57735	0.00791
10471	PFDN6	-0.57626	0.008121
6633	SNRPD2	-0.57554	0.008186
6199	RPS6KB2	-0.57506	0.013675
64210	MMS19	-0.57179	0.008569

(*Continued*)

 © 2024. The Author(s).

Supplementary Table S2 (*Continued*)

EntrezID	Name	CombinedES	Pval
8821	INPP4B	-0.5698	0.01864
7528	YY1	-0.56943	0.00908
23185	LARP4B	-0.56889	0.010784
7169	TPM2	-0.56835	0.011438
26040	SETBP1	-0.56828	0.009308
6687	SPG7	-0.56697	0.009548
29072	SETD2	-0.56594	0.009887
4174	MCM5	-0.56591	0.009721
55746	NUP133	-0.56505	0.009858
5790	PTPRCAP	-0.56461	0.009887
196441	ZFC3H1	-0.56457	0.00982
26953	RANBP6	-0.56434	0.00982
22897	CEP164	-0.5637	0.026138
11055	ZPBP	-0.56229	0.010105
9138	ARHGEF1	-0.55896	0.010735
10277	UBE4B	-0.55741	0.011081
4597	MVD	-0.55722	0.011081
10478	SLC25A17	-0.55671	0.011438
208	AKT2	-0.55529	0.011646
1875	E2F5	-0.55519	0.011646
6636	SNRPF	-0.55487	0.011646
1345	COX6C	-0.55335	0.012017
25950	RWDD3	-0.55026	0.012504
23633	KPNA6	-0.54969	0.01291
2058	EPRS1	-0.54862	0.033539
23492	CBX7	-0.54303	0.014405
3930	LBR	-0.54246	0.014142
3570	IL6R	-0.54057	0.014894
57326	PBXIP1	-0.53966	0.032795
5983	RFC3	-0.53942	0.015328
23250	ATP11A	-0.53885	0.035141
9639	ARHGEF10	-0.53883	0.015063
9761	MLEC	-0.53853	0.015322
6621	SNAPC4	-0.5369	0.015331
23019	CNOT1	-0.5341	0.015991
939	CD27	-0.53366	0.01648
10943	MSL3	-0.53306	0.016317
23081	KDM4C	-0.53269	0.016901
5927	KDM5A	-0.53241	0.01677
6882	TAF11	-0.53205	0.019818
3150	HMGN1	-0.53064	0.016835
5426	POLE	-0.53019	0.01756
8260	NAA10	-0.52964	0.01756

 © 2024. The Author(s).

Supplementary Table S2 (*Continued*)

EntrezID	Name	CombinedES	Pval
23511	NUP188	-0.52896	0.017828
11073	TOPBP1	-0.5279	0.018151
4240	MFGE8	-0.52732	0.018215
30968	STOML2	-0.52658	0.01864
1386	ATF2	-0.52418	0.019178
94239	H2AZ2	-0.52412	0.019225
144699	FBXL14	-0.52185	0.0195
23683	PRKD3	-0.51801	0.021284
1776	DNASE1L3	-0.51714	0.021284
9851	KIAA0753	-0.51592	0.021639
5526	PPP2R5B	-0.51527	0.022315
5310	PKD1	-0.51508	0.021971
7249	TSC2	-0.51437	0.022692
2091	FBL	-0.51339	0.023316
400961	PAIP2B	-0.51243	0.023128
27258	LSM3	-0.51227	0.023555
1819	DRG2	-0.50997	0.024493
23076	RRP1B	-0.50586	0.026138
3275	PRMT2	-0.50357	0.026448
6502	SKP2	-0.5015	0.028483
8533	COPS3	-0.49871	0.029869
10516	FBLN5	-0.49708	0.029691
2800	GOLGA1	-0.49612	0.030136
23262	PPIP5K2	-0.49508	0.030828
221443	OARD1	-0.49479	0.031539
8629	JRK	-0.49192	0.03271
5438	POLR2I	-0.48699	0.035141
22834	ZNF652	-0.48613	0.035428
22872	SEC31A	-0.4855	0.035824
8729	GBF1	-0.48545	0.035612
7571	ZNF23	-0.48475	0.036872
3838	KPNA2	-0.48415	0.036308
51526	OSER1	-0.48346	0.037036
3823	KLRC3	-0.48326	0.036872
4706	NDUFAB1	-0.48308	0.036872
6434	TRA2B	-0.48294	0.036872
23232	TBC1D12	-0.48243	0.037735
3904	LAIR2	-0.48205	0.037036
9918	NCAPD2	-0.47977	0.039589
8330	H2AC15	-0.47934	0.039206
92140	MTDH	-0.47837	0.040562
10762	NUP50	-0.47772	0.040461
3067	HDC	-0.47379	0.043247

(*Continued*)

 © 2024. The Author(s).

Supplementary Table S2 (*Continued*)

EntrezID	Name	CombinedES	Pval
2774	GNAL	-0.47204	0.045453
25875	LETMD1	-0.47098	0.045263
8636	SSNA1	-0.47025	0.045872
9852	EPM2AIP1	-0.46762	0.04715
8225	GTPBP6	-0.46757	0.047382
6637	SNRPG	-0.46757	0.047852
25803	SPDEF	-0.46693	0.047852
57060	PCBP4	-0.46584	0.048715
51232	CRIM1	-0.46512	0.049119
25792	CIZ1	-0.46491	0.04951
25961	NUDT13	-0.46457	0.049119

The table is sorted on the basis of CombinedES.

 © 2024. The Author(s).

Supplementary Table S3 List of hub genes and miRNA on the basis of their hub gene from Protein-Protein interaction of microarray expression profile

EntrezID	Name	Degree (miRNAs)	BetweennessCentrality	ClosenessCentrality	Pval		
1026	CDKN1A	331	0.105685	0.375306	0.000769		
7528	YY1	161	0.035141	0.349681	0.00908		
5928	RBBP4	84	0.008791	0.327406	0.007486		
2064	ERBB2	73	0.012753	0.32911	0.007874		
2353	FOS	58	0.008744	0.331812	0.042176		
7329	UBE2I	18	0.001078	0.299681	0.001474		
3065	HDAC1	10	3.34E-04	0.307148	0.012591		
3066	HDAC2	6	7.41E-05	0.296867	0.002007		
1386	ATF2	6	0.001078	0.299681	0.019178		
5591	PRKDC	2	7.20E-06	0.254929	0.002753		
CDKN1A							
hsa-mir-4537	hsa-mir-520a-3p	hsa-mir-1343-5p	hsa-mir-6779-5p	hsa-mir-4478	hsa-mir-449b-3p	hsa-mir-1207-5p	hsa-mir-101-3p
hsa-mir-2277-3p	hsa-mir-485-5p	hsa-mir-572	hsa-mir-6825-5p	hsa-mir-6874-5p	hsa-mir-6886-3p	hsa-mir-558	hsa-mir-335-5p
hsa-mir-6508-3p	hsa-mir-3668	hsa-mir-6851-5p	hsa-mir-4999-5p	hsa-mir-767-3p	hsa-mir-1260b	hsa-mir-17-5p	hsa-mir-30b-3p
hsa-mir-7110-5p	hsa-mir-4673	hsa-mir-6879-5p	hsa-mir-6819-5p	hsa-mir-6721-5p	hsa-mir-6805-5p	hsa-mir-195-5p	hsa-mir-3180
hsa-mir-938	hsa-mir-5572	hsa-mir-423-3p	hsa-mir-371b-5p	hsa-mir-212-3p	hsa-mir-6778-5p	hsa-mir-4483	hsa-mir-4659a-3p
hsa-mir-4682	hsa-mir-370-3p	hsa-mir-873-3p	hsa-mir-4434	hsa-mir-548m	hsa-mir-196a-5p	hsa-mir-6753-3p	hsa-mir-3663-3p
hsa-mir-6812-5p	hsa-mir-148a-3p	hsa-mir-4268	hsa-mir-3175	hsa-mir-509-3-5p	hsa-mir-4527	hsa-mir-4681	hsa-mir-145-5p
hsa-mir-6883-5p	hsa-mir-892c-5p	hsa-mir-2115-5p	hsa-mir-8078	hsa-let-7a-5p	hsa-mir-363-3p	hsa-mir-519c-3p	hsa-mir-5008-5p
hsa-mir-4322	hsa-mir-3937	hsa-mir-6796-5p	hsa-mir-4769-5p	hsa-mir-3202	hsa-mir-544b	hsa-mir-98-5p	hsa-mir-4650-3p
hsa-mir-515-3p	hsa-mir-4633-3p	hsa-mir-499a-5p	hsa-mir-542-3p	hsa-mir-506-3p	hsa-mir-6501-3p	hsa-mir-133a-3p	hsa-mir-450a-2-3p
hsa-mir-16-5p	hsa-mir-1825	hsa-mir-3617-5p	hsa-mir-4689	hsa-mir-4654	hsa-mir-4773	hsa-mir-4516	hsa-mir-4329
hsa-mir-5685	hsa-mir-6780a-5p	hsa-mir-4455	hsa-mir-576-5p	hsa-mir-6752-5p	hsa-mir-202-3p	hsa-mir-619-3p	hsa-mir-6799-5p
hsa-mir-4257	hsa-mir-6124	hsa-mir-4658	hsa-mir-4487	hsa-mir-1972	hsa-mir-18a-3p	hsa-mir-708-5p	hsa-mir-6753-5p
hsa-mir-15b-5p	hsa-mir-7109-5p	hsa-mir-6887-3p	hsa-mir-4436a	hsa-mir-373-5p	hsa-mir-3169	hsa-mir-939-5p	hsa-mir-519e-3p
hsa-mir-6795-5p	hsa-mir-520b	hsa-mir-7162-3p	hsa-mir-639	hsa-mir-6732-5p	hsa-mir-124-3p	hsa-mir-1909-3p	hsa-mir-616-5p
hsa-mir-1288-3p	hsa-mir-147a	hsa-mir-106b-5p	hsa-mir-345-5p	hsa-mir-5681a	hsa-mir-6803-3p	hsa-mir-6864-3p	hsa-mir-4289
hsa-mir-7151-3p	hsa-mir-6818-5p	hsa-mir-6847-5p	hsa-let-7b-5p	hsa-mir-4254	hsa-mir-6854-5p	hsa-mir-6765-3p	hsa-mir-6880-5p
hsa-mir-6855-5p	hsa-mir-4763-3p	hsa-mir-4700-3p	hsa-mir-15a-5p	hsa-mir-10b-5p	hsa-mir-4632-5p	hsa-mir-3150a-3p	hsa-mir-3180-3p

(*Continued*)

 © 2024. The Author(s).

Supplementary Table S3 (*Continued*)

EntrezID	Name	Degree (miRNAs)	BetweennessCentrality	ClosenessCentrality	Pval		
hsa-mir-132-3p	hsa-mir-1910-3p	hsa-mir-519a-3p	hsa-mir-1237-5p	hsa-mir-5584-5p	hsa-mir-4668-5p	hsa-mir-6887-5p	hsa-mir-642a-3p
hsa-mir-181a-5p	hsa-mir-503-5p	hsa-mir-1229-3p	hsa-mir-1293	hsa-mir-3927-3p	hsa-mir-7704	hsa-mir-20b-5p	hsa-mir-505-5p
hsa-mir-642b-3p	hsa-mir-365a-3p	hsa-mir-8485	hsa-mir-22-3p	hsa-mir-372-3p	hsa-mir-152-3p	hsa-mir-3714	hsa-mir-3689a-3p
hsa-mir-6882-3p	hsa-mir-149-3p	hsa-mir-7107-3p	hsa-mir-519b-3p	hsa-mir-298	hsa-mir-4728-5p	hsa-mir-6815-5p	hsa-mir-4458
hsa-mir-2355-5p	hsa-mir-3689d	hsa-mir-1908-5p	hsa-mir-6500-5p	hsa-mir-4310	hsa-mir-4500	hsa-mir-6816-5p	hsa-mir-3689c
hsa-mir-4267	hsa-mir-95-3p	hsa-mir-6074	hsa-mir-493-5p	hsa-mir-4281	hsa-mir-4418	hsa-let-7f-5p	hsa-mir-637
hsa-mir-6858-5p	hsa-mir-589-3p	hsa-mir-5196-5p	hsa-mir-548e-3p	hsa-mir-4784	hsa-mir-106a-5p	hsa-mir-581	hsa-mir-6740-5p
hsa-mir-4291	hsa-mir-3139	hsa-mir-486-3p	hsa-mir-506-5p	hsa-mir-6885-5p	hsa-mir-615-5p	hsa-mir-574-5p	hsa-mir-328-5p
hsa-mir-3929	hsa-mir-6804-3p	hsa-mir-3689b-3p	hsa-mir-198	hsa-mir-6763-5p	hsa-mir-6832-5p	hsa-let-7c-5p	hsa-mir-5000-3p
hsa-mir-6759-5p	hsa-mir-146a-5p	hsa-mir-641	hsa-mir-4659b-3p	hsa-mir-4643	hsa-mir-583	hsa-mir-6790-5p	hsa-mir-4648
hsa-mir-665	hsa-mir-4717-3p	hsa-mir-4697-5p	hsa-mir-4260	hsa-mir-6841-5p	hsa-mir-519d-3p	hsa-mir-6511a-5p	hsa-mir-4461
hsa-mir-4533	hsa-mir-4290	hsa-mir-6787-5p	hsa-mir-96-5p	hsa-mir-7974	hsa-mir-323a-5p	hsa-mir-567	hsa-mir-578
hsa-let-7d-5p	hsa-mir-942-5p	hsa-mir-7157-5p	hsa-mir-125a-5p	hsa-let-7i-5p	hsa-mir-6802-5p	hsa-mir-6828-3p	hsa-mir-3160-3p
hsa-mir-657	hsa-mir-4505	hsa-mir-6089	hsa-mir-3665	hsa-mir-512-5p	hsa-mir-4724-5p	hsa-mir-5787	hsa-mir-6873-5p
hsa-mir-208b-3p	hsa-mir-1911-3p	hsa-mir-7843-5p	hsa-mir-208a-3p	hsa-mir-6893-3p	hsa-mir-4731-5p	hsa-mir-4419b	hsa-mir-4295
hsa-mir-28-5p	hsa-mir-6842-5p	hsa-mir-1233-5p	hsa-mir-4778-3p	hsa-mir-6737-5p	hsa-mir-509-5p	hsa-mir-4296	hsa-let-7e-5p
hsa-mir-6765-5p	hsa-mir-4645-5p	hsa-mir-663a	hsa-mir-6503-5p	hsa-mir-5703	hsa-mir-4747-5p	hsa-mir-4504	hsa-mir-6801-5p
hsa-mir-3196	hsa-mir-1273h-5p	hsa-mir-6884-5p	hsa-mir-6865-5p	hsa-mir-3910	hsa-mir-3659	hsa-mir-203a-5p	hsa-mir-6752-3p
hsa-mir-4265	hsa-mir-654-3p	hsa-mir-6722-3p	hsa-mir-1537-5p	hsa-mir-4690-3p	hsa-mir-570-3p	hsa-mir-3672	hsa-mir-148b-3p
hsa-mir-302a-3p	hsa-mir-582-5p	hsa-mir-4531	hsa-mir-6761-5p	hsa-mir-520h	hsa-mir-4718	hsa-mir-5571-5p	hsa-mir-6785-5p
hsa-mir-6827-5p	hsa-let-7g-5p	hsa-mir-6735-5p	hsa-mir-365b-3p	hsa-mir-455-3p	hsa-mir-6831-5p	hsa-mir-6749-5p	hsa-mir-6798-5p
hsa-mir-1197	hsa-mir-3616-3p	hsa-mir-5088-3p	hsa-mir-5095	hsa-mir-1248	hsa-mir-93-5p	hsa-mir-645	hsa-mir-4436b-3p
hsa-mir-20a-5p	hsa-mir-299-5p	hsa-mir-4488	hsa-mir-3150b-3p	hsa-mir-4732-5p	hsa-mir-3170	hsa-mir-4719	hsa-mir-1304-5p
hsa-mir-182-5p	hsa-mir-146b-5p	hsa-mir-4721					
YY1							
hsa-mir-30b-3p	hsa-mir-3653-5p	hsa-mir-1208	hsa-mir-93-5p	hsa-mir-149-3p	hsa-mir-19b-3p	hsa-mir-6793-3p	hsa-mir-6780a-5p
hsa-mir-6878-5p	hsa-mir-4789-3p	hsa-mir-548t-5p	hsa-mir-4643	hsa-mir-130b-3p	hsa-mir-378h	hsa-mir-4728-5p	hsa-mir-301b-3p
hsa-mir-6760-3p	hsa-mir-450a-1-3p	hsa-mir-1238-5p	hsa-mir-6779-5p	hsa-mir-543	hsa-mir-3666	hsa-mir-3613-3p	hsa-mir-6799-5p

 © 2024. The Author(s).

Supplementary Table S3 (*Continued*)

EntrezID	Name	Degree (miRNAs)	BetweennessCentrality	ClosenessCentrality	Pval		
hsa-mir-6516-5p	hsa-mir-585-5p	hsa-mir-142-5p	hsa-mir-568	hsa-mir-548g-5p	hsa-mir-6883-5p	hsa-mir-6870-3p	hsa-mir-516b-5p
hsa-mir-3976	hsa-mir-630	hsa-mir-374b-3p	hsa-mir-1468-3p	hsa-mir-1976	hsa-mir-5682	hsa-mir-4638-3p	hsa-mir-3941
hsa-mir-219a-2-3p	hsa-mir-4496	hsa-mir-1299	hsa-mir-4314	hsa-mir-3192-5p	hsa-mir-619-5p	hsa-mir-3122	hsa-mir-221-3p
hsa-mir-3133	hsa-mir-19a-3p	hsa-mir-1909-5p	hsa-mir-193a-3p	hsa-mir-1323	hsa-mir-4658	hsa-mir-219a-1-3p	hsa-mir-4485-5p
hsa-mir-5007-3p	hsa-mir-1281	hsa-mir-4795-3p	hsa-mir-378e	hsa-mir-4522	hsa-mir-5706	hsa-mir-6747-3p	hsa-mir-3177-5p
hsa-mir-3689c	hsa-mir-2467-5p	hsa-let-7c-3p	hsa-mir-331-3p	hsa-mir-34b-3p	hsa-mir-1273h-5p	hsa-mir-8085	hsa-mir-4295
hsa-mir-651-3p	hsa-mir-3679-3p	hsa-mir-4672	hsa-mir-3613-5p	hsa-mir-6840-5p	hsa-mir-491-3p	hsa-mir-6504-3p	hsa-mir-548o-3p
hsa-mir-3155b	hsa-mir-378i	hsa-mir-7-5p	hsa-mir-548f-5p	hsa-mir-2115-5p	hsa-mir-4722-3p	hsa-mir-4782-5p	hsa-mir-30c-1-3p
hsa-mir-6790-5p	hsa-mir-571	hsa-mir-2276-3p	hsa-mir-6513-5p	hsa-mir-892b	hsa-mir-378a-3p	hsa-mir-129-5p	hsa-mir-6788-5p
hsa-mir-4294	hsa-mir-1304-3p	hsa-mir-5588-3p	hsa-mir-548aj-5p	hsa-mir-378f	hsa-mir-34c-5p	hsa-mir-615-3p	hsa-mir-3689a-3p
hsa-mir-30c-2-3p	hsa-mir-431-5p	hsa-mir-5689	hsa-mir-130a-3p	hsa-mir-4778-5p	hsa-mir-378b	hsa-mir-6507-5p	hsa-mir-193b-3p
hsa-mir-34a-5p	hsa-mir-6785-5p	hsa-mir-454-3p	hsa-mir-205-5p	hsa-mir-466	hsa-mir-484	hsa-mir-340-5p	hsa-mir-27b-5p
hsa-mir-6731-5p	hsa-mir-4729	hsa-mir-8066	hsa-mir-6727-3p	hsa-mir-887-5p	hsa-mir-769-3p	hsa-mir-548x-5p	hsa-mir-586
hsa-mir-7106-5p	hsa-mir-139-3p	hsa-mir-4532	hsa-mir-6830-3p	hsa-mir-6890-3p	hsa-mir-6778-3p	hsa-mir-548aw	hsa-mir-548az-5p
hsa-mir-3155a	hsa-mir-5590-3p	hsa-mir-31-5p	hsa-mir-204-3p	hsa-mir-138-1-3p	hsa-mir-6506-5p	hsa-mir-422a	hsa-mir-4279
hsa-mir-511-5p	hsa-mir-301a-3p	hsa-mir-378d	hsa-mir-186-5p	hsa-mir-924	hsa-mir-3913-5p	hsa-mir-5696	hsa-mir-4646-5p
hsa-mir-338-5p	hsa-mir-1279	hsa-mir-455-3p	hsa-mir-3689b-3p	hsa-mir-5582-3p	hsa-mir-378c	hsa-mir-4758-5p	hsa-mir-1247-3p
hsa-mir-183-5p							
RBBP4							
hsa-mir-4284	hsa-mir-3653-5p	hsa-let-7e-5p	hsa-mir-500a-5p	hsa-mir-6780a-5p	hsa-mir-30c-1-3p	hsa-mir-3663-5p	hsa-mir-4680-5p
hsa-mir-423-3p	hsa-mir-6878-5p	hsa-mir-129-5p	hsa-mir-4703-5p	hsa-mir-6513-5p	hsa-mir-7977	hsa-mir-6788-5p	hsa-mir-24-3p
hsa-mir-4294	hsa-mir-3681-3p	hsa-mir-3942-5p	hsa-mir-1228-3p	hsa-mir-4701-5p	hsa-mir-450a-1-3p	hsa-mir-6779-5p	hsa-mir-3689a-3p
hsa-mir-6734-3p	hsa-mir-30c-2-3p	hsa-mir-3187-3p	hsa-mir-431-5p	hsa-mir-6868-3p	hsa-mir-7158-3p	hsa-mir-5092	hsa-mir-383-3p
hsa-mir-216a-3p	hsa-mir-3972	hsa-mir-1202	hsa-mir-6731-5p	hsa-mir-205-5p	hsa-mir-203b-3p	hsa-mir-1183	hsa-mir-887-5p
hsa-mir-6131	hsa-mir-1267	hsa-mir-5006-5p	hsa-mir-640	hsa-mir-6805-3p	hsa-mir-3908	hsa-mir-579-3p	hsa-mir-3122
hsa-mir-4423-5p	hsa-mir-6864-3p	hsa-mir-6767-3p	hsa-mir-4635	hsa-mir-3672	hsa-mir-5691	hsa-mir-588	hsa-mir-3689c
hsa-mir-367-5p	hsa-mir-924	hsa-mir-18a-3p	hsa-mir-3667-3p	hsa-mir-8070	hsa-mir-744-3p	hsa-mir-378a-5p	hsa-mir-548s
hsa-mir-8055	hsa-mir-1273h-5p	hsa-mir-4252	hsa-mir-6501-5p	hsa-mir-8085	hsa-mir-3194-3p	hsa-mir-5696	hsa-mir-3913-5p
hsa-mir-128-3p	hsa-mir-6759-3p	hsa-mir-429	hsa-mir-5693	hsa-mir-3689b-3p	hsa-mir-664b-3p	hsa-mir-342-3p	hsa-mir-324-5p
hsa-mir-4435	hsa-mir-4766-3p	hsa-mir-30b-3p	hsa-mir-4755-3p				

(*Continued*)

© 2024. The Author(s).

Supplementary Table S3 (*Continued*)

EntrezID	Name	Degree (miRNAs)	BetweennessCentrality	ClosenessCentrality	Pval		
ERBB2							
hsa-mir-199b-5p	hsa-mir-3921	hsa-mir-146a-3p	hsa-mir-125a-5p	hsa-mir-6885-5p	hsa-mir-130b-3p	hsa-mir-4680-3p	hsa-mir-552-3p
hsa-mir-4427	hsa-mir-4677-5p	hsa-mir-548d-3p	hsa-mir-323b-5p	hsa-mir-21-5p	hsa-mir-125b-5p	hsa-mir-7845-5p	hsa-mir-6795-5p
hsa-mir-670-3p	hsa-mir-329-5p	hsa-mir-6734-3p	hsa-mir-4790-3p	hsa-mir-6868-3p	hsa-mir-1296-5p	hsa-mir-3127-5p	hsa-mir-7109-5p
hsa-mir-199a-5p	hsa-mir-7156-5p	hsa-mir-4790-5p	hsa-mir-155-3p	hsa-mir-4464	hsa-mir-2116-5p	hsa-mir-34a-5p	hsa-mir-4441
hsa-mir-6754-5p	hsa-mir-193a-5p	hsa-mir-593-3p	hsa-mir-6891-3p	hsa-mir-888-3p	hsa-mir-4748	hsa-mir-22-5p	hsa-mir-205-5p
hsa-mir-22-3p	hsa-mir-3140-3p	hsa-mir-134-5p	hsa-mir-4270	hsa-mir-559	hsa-mir-193a-3p	hsa-mir-5187-3p	hsa-mir-25-3p
hsa-mir-486-3p	hsa-mir-124-3p	hsa-mir-4653-5p	hsa-mir-1254	hsa-mir-375	hsa-mir-328-5p	hsa-mir-301a-5p	hsa-mir-541-3p
hsa-mir-331-3p	hsa-mir-6887-5p	hsa-mir-4315	hsa-mir-6730-3p	hsa-mir-4506	hsa-mir-3622b-5p	hsa-mir-326	hsa-mir-3667-3p
hsa-mir-211-3p	hsa-mir-4326	hsa-mir-6739-3p	hsa-mir-3116	hsa-mir-6072	hsa-mir-133a-3p	hsa-mir-498	hsa-mir-301b-5p
hsa-mir-4273							
FOS							
hsa-mir-5095	hsa-mir-7-5p	hsa-mir-222-3p	hsa-mir-548v	hsa-mir-196b-5p	hsa-mir-34a-5p	hsa-mir-5586-5p	hsa-mir-101-3p
hsa-mir-4726-5p	hsa-mir-29a-3p	hsa-mir-1292-3p	hsa-mir-146a-5p	hsa-mir-5581-3p	hsa-mir-6077	hsa-mir-619-5p	hsa-mir-449a
hsa-mir-4530	hsa-mir-19b-2-5p	hsa-mir-29c-3p	hsa-mir-181b-5p	hsa-mir-770-5p	hsa-mir-155-5p	hsa-mir-6885-3p	hsa-mir-181a-5p
hsa-mir-19a-5p	hsa-mir-338-3p	hsa-mir-323b-3p	hsa-mir-8083	hsa-mir-4640-5p	hsa-mir-5089-3p	hsa-mir-6854-3p	hsa-mir-6508-3p
hsa-mir-1234-3p	hsa-mir-3065-3p	hsa-mir-139-5p	hsa-mir-29b-3p	hsa-mir-221-3p	hsa-mir-6816-3p	hsa-mir-8080	hsa-mir-6872-3p
hsa-mir-490-5p	hsa-mir-19b-1-5p	hsa-mir-187-5p	hsa-mir-4438	hsa-mir-3622b-5p	hsa-mir-543	hsa-mir-6504-3p	hsa-mir-8081
hsa-mir-627-5p	hsa-mir-335-5p	hsa-mir-937-5p	hsa-mir-4733-3p	hsa-mir-215-5p	hsa-mir-7107-5p	hsa-mir-101-5p	hsa-mir-192-5p
hsa-mir-493-5p	hsa-mir-7151-3p						
UBE2I							
hsa-mir-324-5p	hsa-mir-4426	hsa-mir-30c-5p	hsa-mir-4707-3p	hsa-mir-361-3p	hsa-mir-195-3p	hsa-mir-7108-3p	hsa-mir-652-3p
hsa-mir-30e-5p	hsa-mir-214-3p	hsa-mir-4662b	hsa-mir-4647	hsa-mir-200c-3p	hsa-mir-6789-3p	hsa-mir-1-3p	hsa-let-7b-5p
hsa-mir-188-5p	hsa-mir-566						
HDAC1							
hsa-mir-24-3p	hsa-mir-874-3p	hsa-mir-34a-5p	hsa-mir-449a	hsa-mir-30a-5p	hsa-mir-671-5p	hsa-mir-615-3p	hsa-mir-92a-3p
hsa-mir-520h	hsa-mir-449b-5p						

 © 2024. The Author(s).

Supplementary Table S3 (*Continued*)

EntrezID	Name	Degree (miRNAs)	BetweennessCentrality	ClosenessCentrality	Pval		
HDAC2							
hsa-mir-1-3p	hsa-mir-92a-3p	hsa-let-7f-5p	hsa-mir-145-5p	hsa-mir-149-5p	hsa-mir-455-3p		
ATF2							
hsa-mir-15a-5p	hsa-mir-374b-5p	hsa-mir-19b-3p	hsa-mir-335-5p	hsa-mir-204-3p			
PRKDC							
hsa-mir-218-5p	hsa-mir-101-5p						

The Degree defined as the number of connections to the other nodes. Here the node is represented to miRNAs.

 © 2024. The Author(s).

Supplementary Table S4 List of miRNA that are differentially expressed in all the inflammatory diseases and pulmonary embolism with individual Fold change

miRNAs ID	Fold-Change			
	PE	RA	SLE	IBD
hsa-mir-107	3.287293	1.221048	0.964464	1.02506
hsa-mir-133b	3.886146	1.072266	0.986575	1.066008
hsa-mir-137	5.798501	1.004815	1.350974	0.978116
hsa-mir-184	1.843209	0.982258	0.986575	0.987014
hsa-mir-147b	2.732402	1.036071	0.986575	1.037874
hsa-mir-198	3.886146	1.034082	0.986575	0.979168
hsa-mir-217	3.886146	1.039182	0.986575	0.972393
hsa-mir-298	3.886146	0.957452	0.986575	0.985637
hsa-mir-301b	3.462418	1.047344	0.986575	1.090029
hsa-mir-326	3.954184	1.091438	0.790041	1.072459
hsa-mir-346	4.837796	0.968984	0.986575	1.067754
hsa-mir-325	3.886146	0.960149	0.986575	0.974953
hsa-mir-384	3.886146	1.005983	0.986575	1.002657
hsa-mir-422a	4.241281	0.874504	0.986575	1.168442
hsa-mir-429	4.361396	1.052004	0.986575	0.995388
hsa-mir-375	1.83626	0.991237	0.986575	0.996145
hsa-mir-449a	3.886146	1.001138	1.401527	1.048209
hsa-mir-484	1.724825	1.079497	1.043912	1.04239
hsa-mir-492	3.886146	0.969796	0.986575	1.091079
hsa-mir-448	3.886146	0.997419	0.986575	0.99912
hsa-mir-496	3.886146	1.056884	0.986575	1.169624
hsa-mir-507	2.748263	1.035633	0.986575	0.98008
hsa-mir-520b	3.868167	1.051426	0.986575	1.008213
hsa-mir-518b	4.126876	0.950399	0.986575	0.9198
hsa-mir-520e	3.748231	1.001692	0.986575	1.007513
hsa-mir-636	2.256708	0.998961	0.939718	1.223337
hsa-mir-890	3.886146	0.996465	0.986575	1.183095
hsa-mir-618	2.169526	0.964669	0.986575	0.979089
hsa-mir-891b	3.886146	1.002327	0.986575	1.010898
hsa-mir-892a	3.886146	1.017097	0.986575	1.015623

Note: Grey colors are up-regulated DEmiRs and salmon pink is down-regulated DEmiRs.

 © 2024. The Author(s).
